# Dissecting pleiotropy between major depressive disorder and physical disease comorbidities

**DOI:** 10.1038/s41588-026-02735-3

**Published:** 2026-08-26

**Authors:** Damian J. Woodward, William R. Reay, Briar Wormington, Santiago Diaz-Torres, Jue-Sheng Ong, Zachary F. Gerring, Jackson G. Thorp, Eske M. Derks

**Affiliations:** 1 Brain and Mental Health, QIMR Berghofer, Brisbane, Queensland Australia; 2 School of Biomedical Science, Queensland University of Technology, Brisbane, Queensland Australia; 3 Menzies Institute for Medical Research, University of Tasmania, Hobart, Tasmania Australia; 4 School of Biomedical Science, University of Queensland, Brisbane, Queensland Australia; 5 Global Precision Health Lab, Population Health Program, QIMR Berghofer, Brisbane, Queensland Australia; 6 Genetics and Gene Regulation, Walter and Eliza Hall Institute of Medical Research, Melbourne, Victoria Australia

**Keywords:** Genome-wide association studies, Depression, Functional genomics

## Abstract

Major depressive disorder (MDD) is characterized by substantial comorbidity with medical conditions. To achieve better outcomes for patients with MDD, an improved understanding of the mechanisms underlying pervasive comorbidities is required. Here, to this end, we mapped patterns of pleiotropy by defining four clusters of physical diseases (cardiovascular, metabolic, gastrointestinal and immune) and analyzed their genetic relationships with MDD using genomic structural equation modeling. Three disease clusters exhibited independent associations with MDD and accounted for 47% of MDD *h*^2^_SNP_, with the gastrointestinal disease cluster having the strongest association (*β* = 0.63, s.e. = 0.05, *P* = 3.04 × 10^−30^). In addition, we identified independent loci associated with the shared genetic liability between each disease cluster and MDD, revealing different pleiotropic components. Characterization of these loci revealed previously unidentified associations with MDD and physical disease traits, along with unique biological pathways, drug groups, cell types and genes associated with each disease–MDD cluster. Our findings reveal genetic connections implicating the gut–brain axis as a key mechanism underlying the comorbidity of physical diseases in MDD. This work advances our understanding of MDD by highlighting unique and shared genetic components across different disease systems.

## Abstract (web summary)

This study uses genomic structural equation modeling to analyze genetic relationships between major depressive disorder and physical disease comorbidities and identifies independent loci associated with their shared genetic liability.

## Main

Major depressive disorder (MDD) is characterized by considerable variation in symptoms and clinical presentations, including associations with a diverse range of medical conditions that frequently co-occur^1^. MDD ranks as the second leading cause of years lived with disability^2^, with over one-third of the total nonfatal burden among patients with MDD attributed to physical disease comorbidity^3^. Patients with MDD are at greater risk of suffering from a variety of medical diseases^4^ (for example, cardiovascular or inflammatory), which have been shown to decrease adherence to medical treatment^5,6^, worsen clinical outcomes^7^ and increase mortality^8^.

At a phenotypic level, MDD is associated with various diseases that can collectively be grouped into physical disease clusters. Shifting the focus to investigating these clusters can identify shared underlying risk factors across these diseases and their combined association with MDD. For example, MDD has an association with diseases relating to cardiovascular health, increasing the risk of future cardiovascular events^9,10^ and high comorbidity rates across cardiovascular diseases (CVD)^11,12^. Additionally, metabolic dysfunction has been implicated in patients with MDD with metabolic syndrome^13^ (that is, the clustering of metabolic risk factors), diabetes mellitus^14^ and lipid and glucose levels^15^. Gastrointestinal health is progressively being linked to psychiatric health^16^, and gastrointestinal diseases have a higher prevalence among patients with MDD^17,18^. Mechanisms of the immune system have been connected to MDD, with low-grade inflammation observed in some cases of MDD^19^ and certain immune disorders are associated with a history of depression^20^. Notably, these systems do not operate in isolation and have complex interactions. It is important to examine the influence of these disease groups on MDD, both individually and collectively.

The sharing of genetic risk factors (pleiotropy) may be one factor underlying the relationship between phenotypic comorbidities. A genome-wide association study (GWAS) of MDD found that the proportion of heritability explained by common genetic variants (*h*^2^_SNP_) was approximately 7%, with 243 associated loci identified^21^. Part of this genetic variation has exhibited overlap with physical diseases. MDD has shown low to moderate genetic correlations with disorders among physical disease clusters, including cardiovascular^22^, metabolic^23^, gastrointestinal^24^ and immune-related conditions^25^. Dissecting MDD *h*^2^_SNP_ can provide a framework to examine different pleiotropic components. For example, ref. ^26^ identified genetic drivers of type 2 diabetes heterogeneity by decomposing GWAS association signals using cardiometabolic phenotypes. This enabled the characterization of variant clusters to determine enrichment for specific cells and pathways, highlighting diverse etiological pathways driving a complex disease. Using genetic data to partition MDD pleiotropy can be a promising approach to identifying the genetic drivers of physical disease comorbidity, potential shared etiology, subgroups with similar clinical features, and improved treatment approaches.

Large-scale GWASs have introduced new opportunities to investigate the genetic interplay between disease systems. The multivariable method, genomic structural equation modeling (SEM), can be used to model traits using GWAS summary statistics and genetic correlations to capture a disease group’s shared genetic liability via latent factors^27^. For example, ref. ^22^ identified a cardiovascular-derived latent factor and determined that MDD and CVD share part of the genetic overlap with immune and metabolic risk factors, with metabolic factors partially explaining the causal effects of the genetic component of MDD on cardiovascular risk. That study focused on a CVD latent factor, and the application of this modeling framework to other disease systems has yet to be explored. Further modeling of genetic connections across physical disease clusters can improve our understanding of how genetic predispositions contribute to various comorbid conditions, their independent effects on MDD and the characterization of shared genetic liability between MDD and each disease group.

**Fig. 1:**
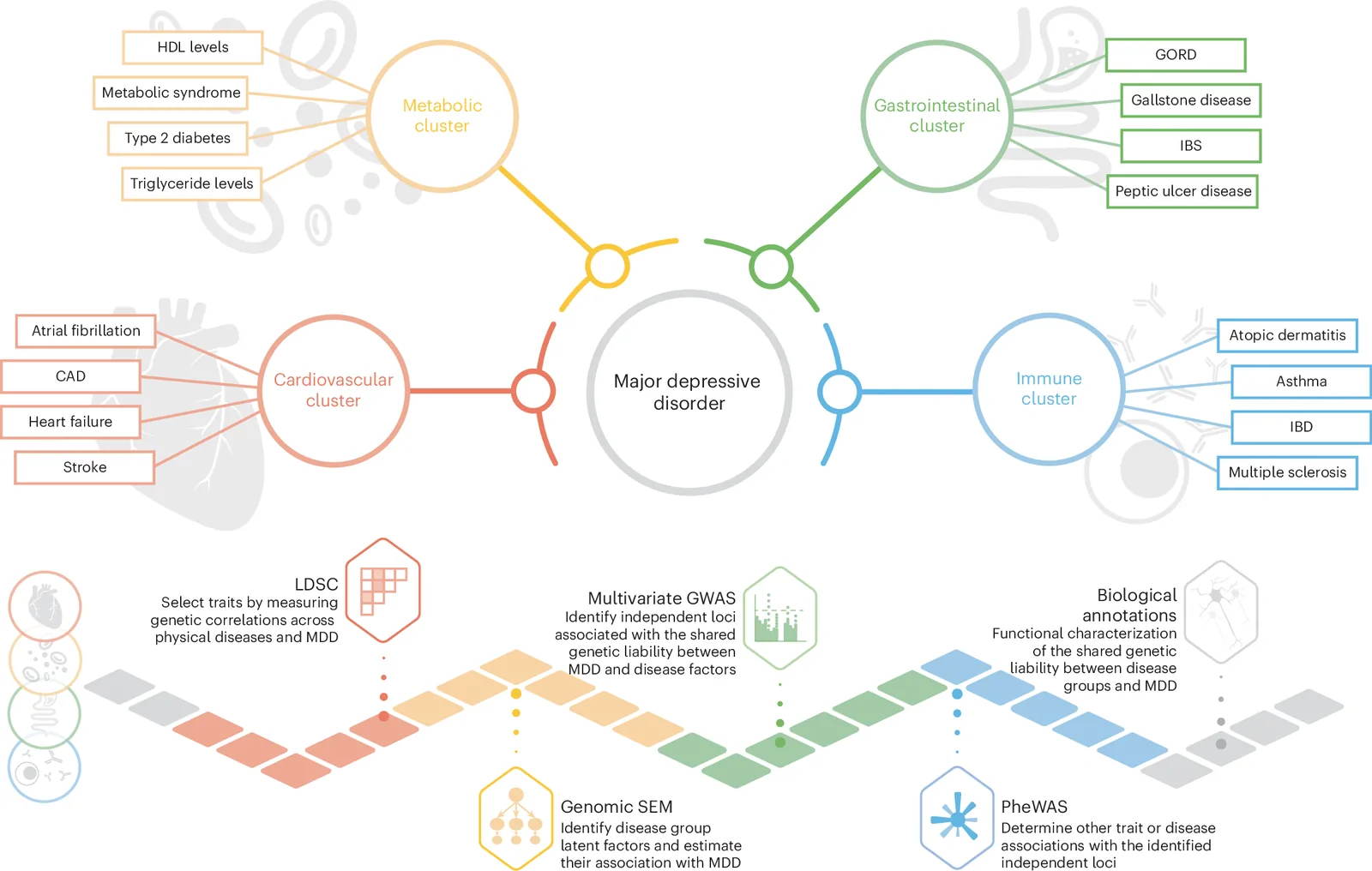
Overview of the study design and workflow. The selected traits for each physical disease cluster are highlighted and each major step in the workflow is outlined. Cardiovascular cluster, red; metabolic cluster, yellow; gastrointestinal cluster, green; immune cluster, blue. CAD, coronary artery disease; IBS, irritable bowel syndrome; IBD, inflammatory bowel disease; HDL, high-density lipoprotein cholesterol.

Here we investigated pleiotropic components between MDD and physical disease clusters and explored their downstream effects to identify genetic drivers of MDD–physical disease comorbidity. We defined physical diseases as nonpsychiatric medical conditions and examined four physical disease clusters (cardiovascular, metabolic, gastrointestinal and immune). These were chosen based on previous research^22,24,28,29^ and reflect common physical comorbidity groups encompassing a range of disease systems (Fig. 1). We aimed to (1) identify disease cluster latent factors associated with MDD and measure their independent effects on the genetic variance of MDD; (2) capture and characterize the shared genetic variance between MDD and each disease cluster.

## Results

### Modeling the genetic covariance between MDD and physical disease clusters

**Fig. 2:**
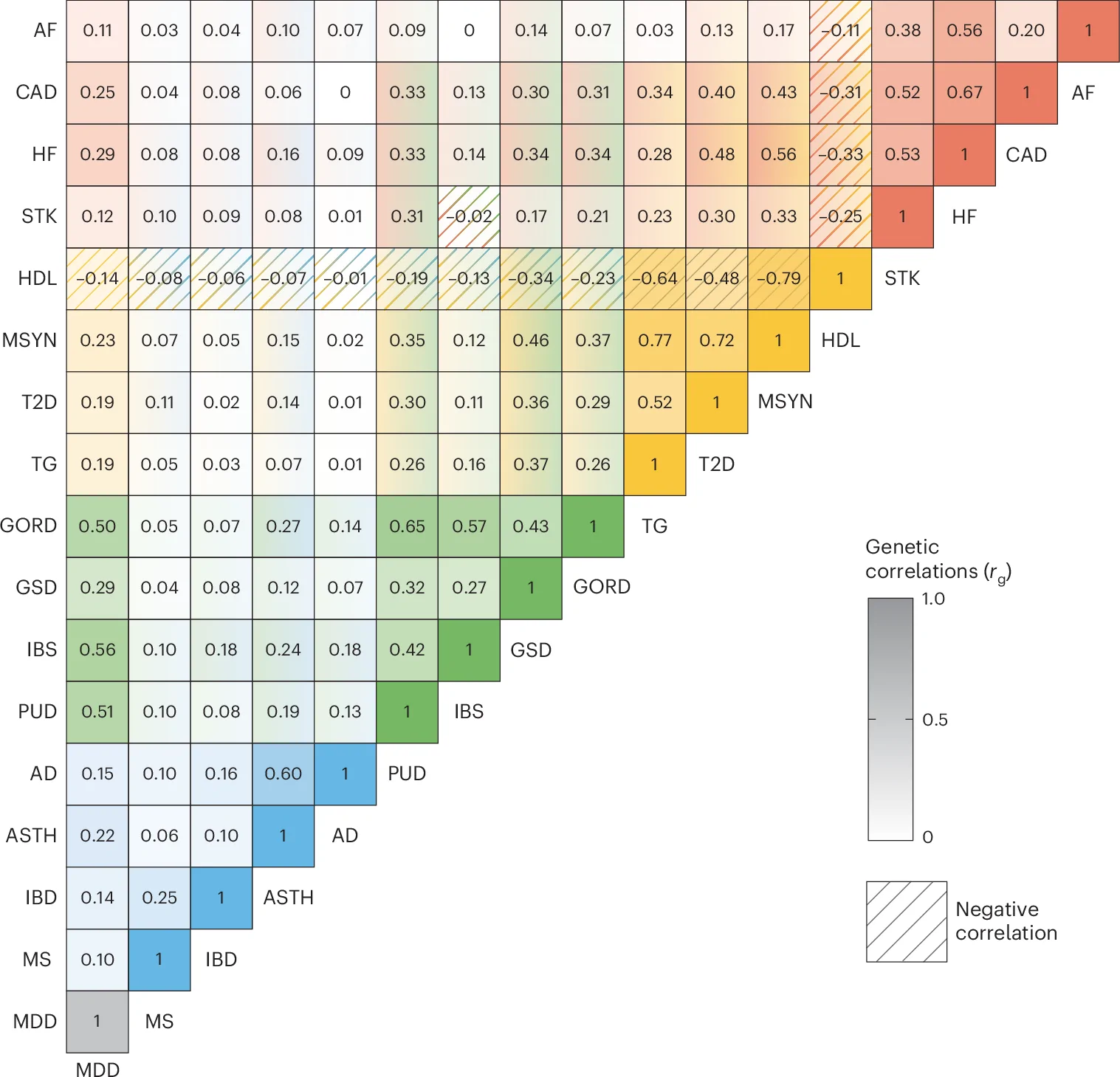
Heatmap depicting genome-wide correlations (*r*_g_) of traits in disease clusters and MDD using LDSC estimated from common variants. Heatmap shows traits that were significantly correlated with MDD after Bonferroni correction (two-sided *P* < 0.05/19 traits). Cardiovascular cluster, red; metabolic cluster, yellow; gastrointestinal cluster, green; immune cluster, blue. AF, atrial fibrillation; HF, heart failure; STK, stroke; MSYN, metabolic syndrome; T2D, type 2 diabetes; TG, triglycerides; GSD, gallstone disease; PUD, peptic ulcer disease; AD, atopic dermatitis; ASTH, asthma; MS, multiple sclerosis. Source data

We compiled a broad set of relevant traits across the four clusters, drawing on previous research linking traits to disease clusters and MDD^29^ (‘Data selection’ in Supplementary Results). We selected well-powered traits (*h*^2^_SNP_ *z* > 7) for each disease cluster using linkage disequilibrium score regression (LDSC). Of these, 16 showed a significant genetic correlation with MDD after correcting for multiple testing across 19 traits (α < 2.63 × 10^−3^; 0.05/19) and were retained for downstream analysis (Fig. 2 and Table 1). MDD had the largest correlations on average with gastrointestinal traits (mean |*r*_g_| = 0.47; range = 0.29–0.56), followed by cardiovascular traits (mean |*r*_g_| = 0.19; range = 0.11–0.29), metabolic traits (mean |*r*_g_| = 0.19; range = −0.14–0.23) and immune traits (mean |*r*_g_| = 0.15; range = 0.10–0.22).

**Table 1:** Traits identified for disease groups used for latent factor analysis and their genetic correlation (*r*_g_) with MDD

Disorder or trait	Abbreviation	Sample size	Cases	Controls	*h*^2^_SNP_	*h*^2^_SNP_ *z* score	MDD *r*_g_	*r*_g_ *P* value
Atrial fibrillation	AF	1,030,836	60,620	970,216	0.064	12.70	0.11	1.79 × 10^−7^
Coronary artery disease	CAD	1,165,690	181,522	984,168	0.054	17.80	0.25	1.99 × 10^−42^
Heart failure	HF	977,323	47,309	930,014	0.029	12.50	0.29	4.57 × 10^−23^
Stroke	STK	446,696	40,585	406,111	0.030	9.04	0.12	2.51 × 10^−4^
High-density lipoprotein cholesterol	HDL	1,320,016	NA	NA	0.101	14.60	−0.14	2.85 × 10^−21^
Metabolic syndrome	MSYN	291,107	59,677	231,430	0.195	18.90	0.23	1.80 × 10^−27^
Type 2 diabetes	T2D	1,812,017	242,283	1,569,734	0.129	22.80	0.19	2.69 × 10^−28^
Triglycerides	TG	1,320,016	NA	NA	0.087	11.00	0.19	2.32 × 10^−32^
Gastroesophageal reflux disease	GORD	456,327	54,854	401,473	0.082	19.60	0.50	7.26 × 10^−66^
Gallstone disease	GSD	550,437	43,639	506,798	0.088	11.40	0.29	5.42 × 10^−32^
Irritable bowel syndrome	IBS	486,601	53,400	433,201	0.073	17.30	0.56	1.10 × 10^−83^
Peptic ulcer disease	PUD	456,327	16,666	439,661	0.073	8.13	0.51	9.49 × 10^−38^
Atopic dermatitis	AD	864,982	60,653	804,329	0.055	7.18	0.15	1.67 × 10^−9^
Asthma	ASTH	303,859	64,538	239,321	0.143	13.60	0.22	1.51 × 10^−30^
Inflammatory bowel disease	IBD	59,957	25,042	34,915	0.152	11.60	0.14	1.23 × 10^−7^
Multiple sclerosis	MS	41,505	14,802	26,703	0.115	11.40	0.10	2.94 × 10^−5^

NA, not applicable.

**Extended Data Fig. 1:**
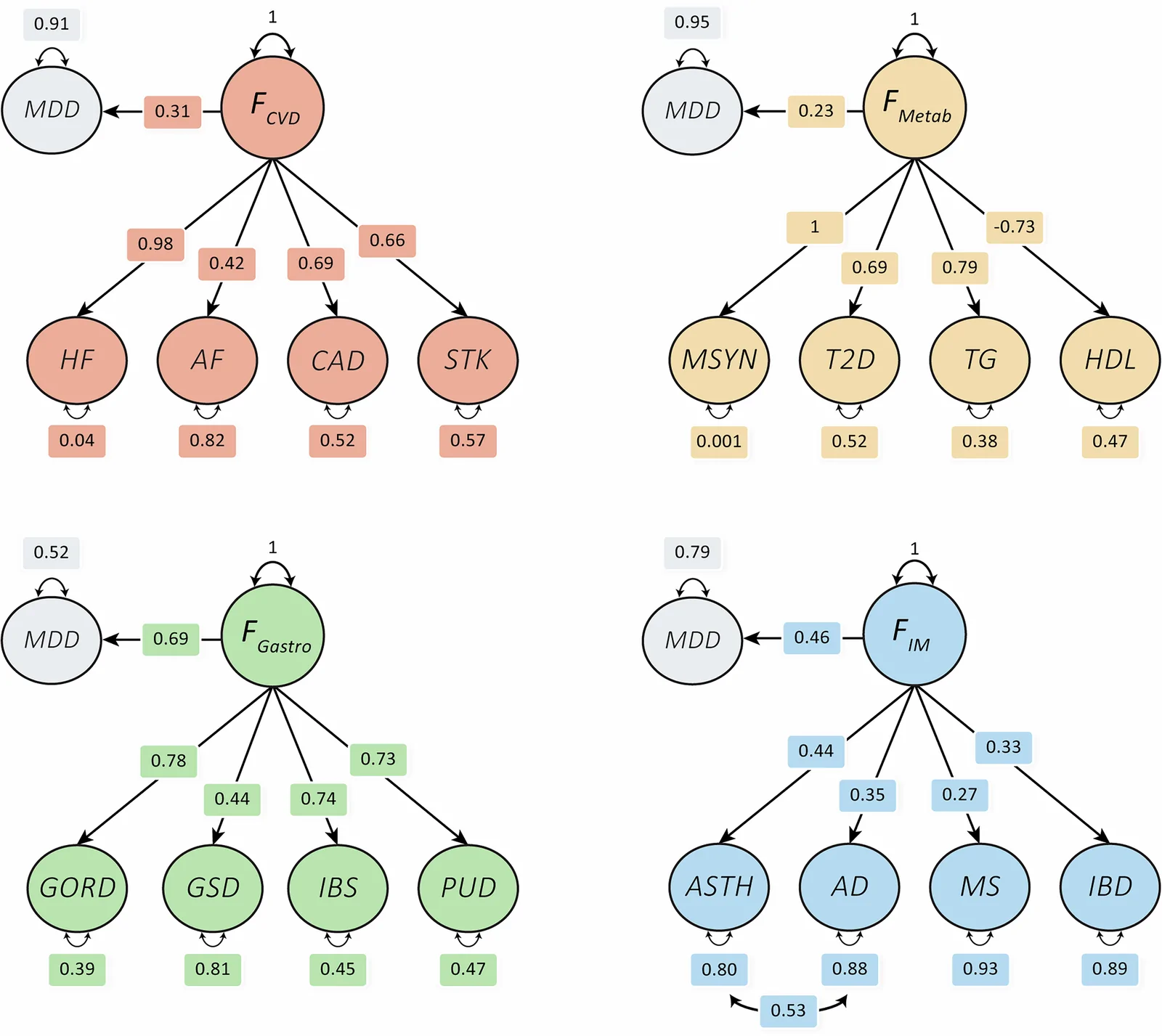
Common factor models for physical disease traits and their combined association with major depressive disorder (MDD). Each latent factor (F) captures the shared genetic variance across its underlying traits, with MDD regressed onto the factor. Boxed values represent standardized estimated parameters. AF, atrial fibrillation; CAD, coronary artery disease; HF, heart failure; STK, stroke; HDL, high-density lipoprotein cholesterol; MSYN, metabolic syndrome; T2D, type 2 diabetes; TG, triglycerides; GORD, gastroesophageal reflux disease; GSD, gallstone disease; IBS, irritable bowel syndrome; PUD, peptic ulcer disease; AD, atopic dermatitis; ASTH, asthma; IBD, inflammatory bowel disease; MS, multiple sclerosis. Source data

Next, we used genomic SEM^27^ to estimate latent factors for each of the four disease clusters (representing the genetic liability shared across traits within each cluster) and measure their association with MDD (Extended Data Fig. 1 and ‘Disease–MDD latent factor analysis’ in Supplementary Results; for *h*^2^_SNP_ *z* > 4, see ‘Disease–MDD latent factor sensitivity analysis (SNP-based heritability *z* > 4)’ in Supplementary Results). Each disease latent factor had a significant association with MDD, with the gastrointestinal factor having the strongest (gastrointestinal, *β* = 0.69, s.e. = 0.03, *P* = 5.87 × 10^−158^, *R*^2^ = 0.48; immune, *β* = 0.46, s.e. = 0.06, *P* = 1.64 × 10^−16^, *R*^2^ = 0.21; cardiovascular, *β* = 0.31, s.e. = 0.02, *P* = 5.12 × 10^−46^, *R*^2^ = 0.09; metabolic, *β* = 0.23, s.e. = 0.02, *P* = 6.55 × 10^−44^, *R*^2^ = 0.05).

**Fig. 3:**
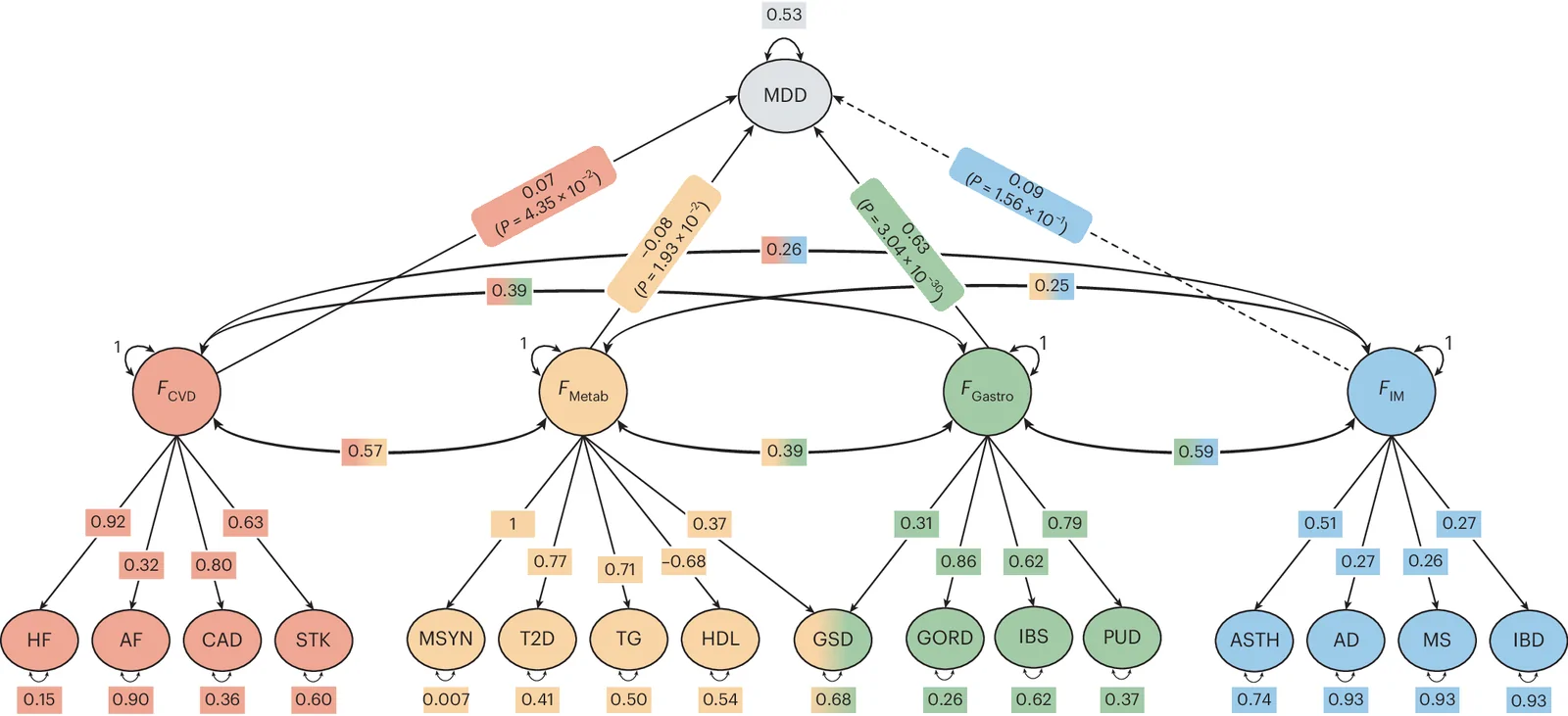
Multiple regression model of disease group latent factors (*F*) and their independent associations with MDD estimated using genomic SEM. A solid line indicates a significant independent association (*P* < 0.05) and a dotted line indicates an insignificant association; path significance tested two-sided. Boxed values represent estimated parameters using standardized estimates (Supplementary Table 3; STD_All column). Model fit—CFI = 0.918; SRMR = 0.055. Cardiovascular cluster, red; metabolic cluster, yellow; gastrointestinal cluster, green; immune cluster, blue. Metab, metabolic; gastro, gastrointestinal; IM, immune. Source data

Combining these, we developed a four-factor model to identify their independent associations with MDD (Fig. 3 and Supplementary Table 3; comparative fit index (CFI) = 0.918; standardized root mean square residual (SRMR) = 0.055). When jointly modeled, we observed that the gastrointestinal cluster had a strong independent association with MDD, albeit weaker than the individual model (*β* = 0.63, s.e. = 0.05, *P* = 3.04 × 10^−30^). The cardiovascular (*β* = 0.07, s.e. = 0.04, *P* = 0.04) and metabolic (*β* = −0.08, s.e. = 0.03, *P* = 0.02) associations with MDD were attenuated but still demonstrated small independent associations. The immune factor showed a nonsignificant association when controlling for the other disease clusters (*β* = 0.09, s.e. = 0.06, *P* = 0.156). Together, the four factors explained 47% of the *h*^2^_SNP_ of MDD (*R*^2^ = 0.47). The combined model did not explain more variance than the individual gastrointestinal model, indicating overlapping genetic contributions across factors. These results suggest that the gastrointestinal cluster has the strongest association with MDD and largely accounts for the genetic variance of the remaining disease clusters to MDD.

### Capturing the shared genetic liability between MDD and disease clusters

**Fig. 4:**
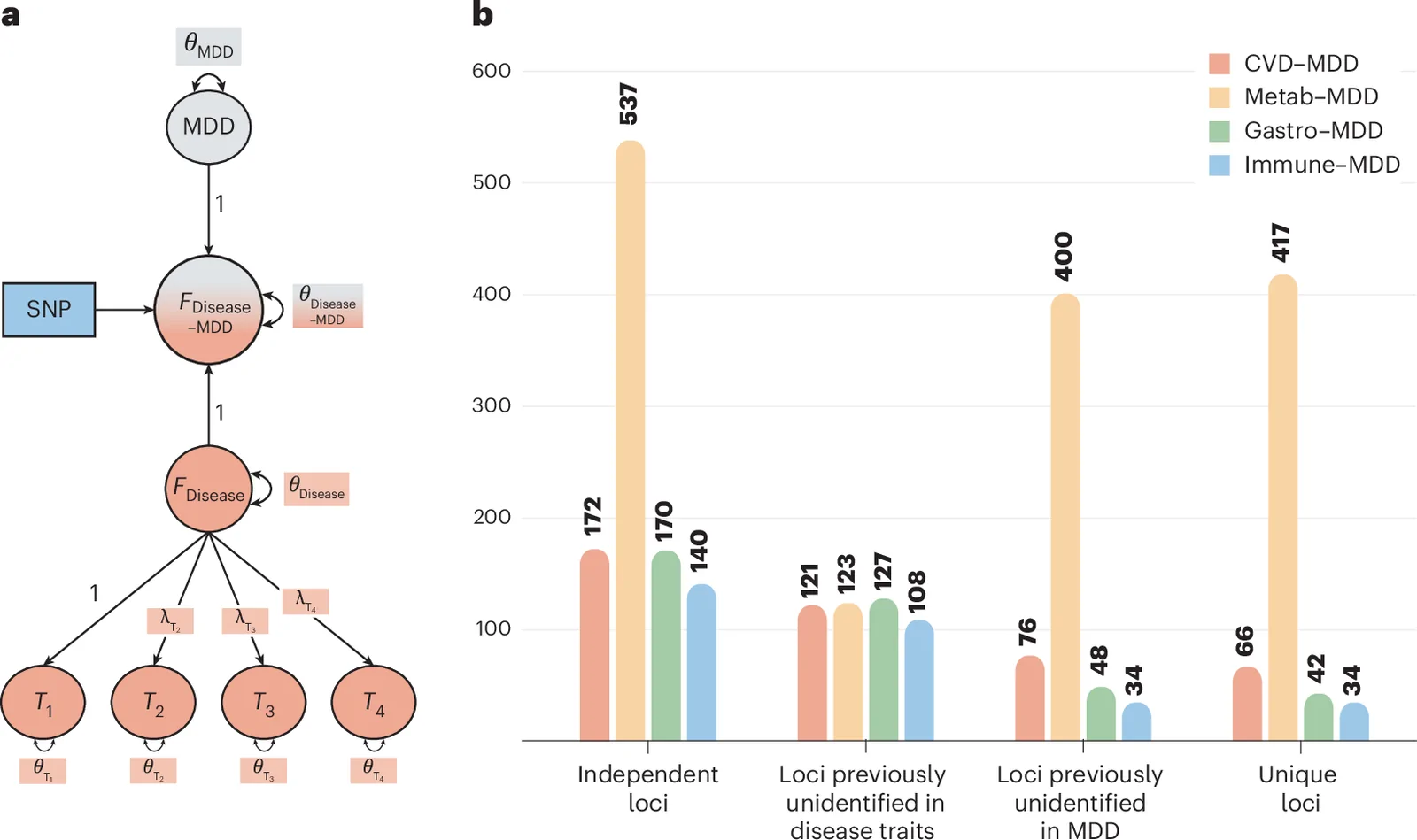
Multivariate GWAS of second-order disease–MDD latent factors estimated using genomic SEM. **a**, Second-order factor (*F*_Disease–MDD_) model for multivariate GWAS capturing shared genetic liability between MDD and disease factor (*F*_Disease_) with unstandardized factor loadings constrained to be equal for model identification. **b**, Number of independent loci associated with the second-order factor disease models estimated using FUMA (*r*^2^ > 0.6, LD block distance > 250 kb), further partitioned into the number of loci previously unidentified for disease traits, previously unidentified for MDD, and unique to the disease–MDD factor. Cardiovascular cluster, red; metabolic cluster, yellow; gastrointestinal cluster, green; immune cluster, blue. Source data

We aimed to functionally characterize each cluster’s shared component with MDD to identify pleiotropic loci, biological mechanisms and secondary risk pathways underlying MDD–physical disease comorbidity. To capture the shared genetic component between MDD and each disease cluster, we estimated models in genomic SEM in which a second-order latent factor loaded onto both MDD and the lower-order disease factor (‘disease’–MDD factor; Fig. 4a and ‘Second-order latent factor models’ in Supplementary Results). We then conducted a GWAS on each disease–MDD latent factor to identify pleiotropic variants between the disease cluster and MDD. To ensure that loci associated with the disease–MDD factors were not driven primarily by a single trait, we excluded variants with highly discordant effects (*Q*_SNP_ *P* < 5 × 10^−8^; ‘*Q*_SNP_ heterogeneity test’ in Supplementary Results).

Figure 4b illustrates the independent risk loci identified from the multivariate GWAS for each disease–MDD factor (*r*^2^ = 0.60; maximum distance between MDD blocks ≥250 kb; Supplementary Tables 15–18). Loci were classified into those previously unidentified for MDD and disease cluster traits. These loci may indicate specific mechanisms more closely related to the shared genetic variance between disease clusters and MDD rather than to individual traits. Additionally, we compared loci between each disease–MDD factor to identify those unique to each factor. These loci may indicate disease cluster-specific genetic drivers of MDD comorbidity.

First, the metabolic–MDD factor had the highest number of independent loci, with 537 association signals, of which 417 were unique to this factor (not observed in the other factors). The large number of loci in this cluster highlights the statistical power of the univariate metabolic GWAS (‘Power quantification’ in Supplementary Results). Next, 172 independent loci were identified for the CVD–MDD factor. Of these loci, 66 were unique to the CVD–MDD factor. For the gastrointestinal–MDD factor, 170 independent loci were associated, including 42 unique loci. Lastly, the immune–MDD factor had the smallest number of independent loci, with 141. This was likely restricted by the low variance captured within the immune latent factor, as the weak genetic correlations among immune traits hindered the boost of effective sample size in this model. Of the identified risk loci for the immune–MDD factor, 34 were unique.

We also performed phenome-wide association studies (PheWASs) to determine diseases and traits associated with the independent loci using the Open Targets Genetics platform^30^. These results were largely confirmatory, with strong associations observed for psychiatric traits and known risk factors for each disease cluster, supporting that the captured variance was linked to MDD and the disease cluster (‘PheWAS’ in Supplementary Results). For example, the CVD–MDD second-order factor had high counts for anthropometric measures and psychiatric and cardiovascular traits. Additionally, we examined the association between each cluster and MDD while controlling for social and behavioral traits (‘Social and behavioral traits’ in Supplementary Results). Overall, we observed that the gastrointestinal cluster captures shared components linking social and behavioral patterns to MDD liability, including physical activity, sleep duration, alcohol use and high income.

### Investigating the association between the gastrointestinal cluster and MDD

Given the strong correlations between MDD and the gastrointestinal cluster, the high MDD variance explained by the gastrointestinal factor and the strong psychiatric associations observed in the PheWAS (‘Gastrointestinal–MDD PheWAS’ in Supplementary Results), this factor was hypothesized to capture psychophysiological mechanisms underlying gut–brain axis dysregulation. These findings are consistent with the observation that psychological factors (for example, stress, anxiety or depression) influence gastrointestinal function and symptoms^31,32^. Notably, some gastrointestinal traits, including gastroesophageal reflux disease (GORD), can be separated into functional and nonfunctional subgroups. Functional GORD is a disorder subgroup associated with GORD symptoms but with the absence of pathological acid damage along the esophageal tract^33^. Therefore, we further investigated the relationship between functional gastrointestinal disorders and the second-order factor using GORD subgroup GWASs (‘Gastrointestinal factor follow-up analysis’ in Supplementary Results). Overall, this follow-up analysis demonstrated that the second-order factor was primarily associated with functional GORD, indicating that the captured variance likely reflects psychophysiological mechanisms linked to the gut–brain axis rather than gastrointestinal tract pathology.

**Extended Data Fig. 2:**
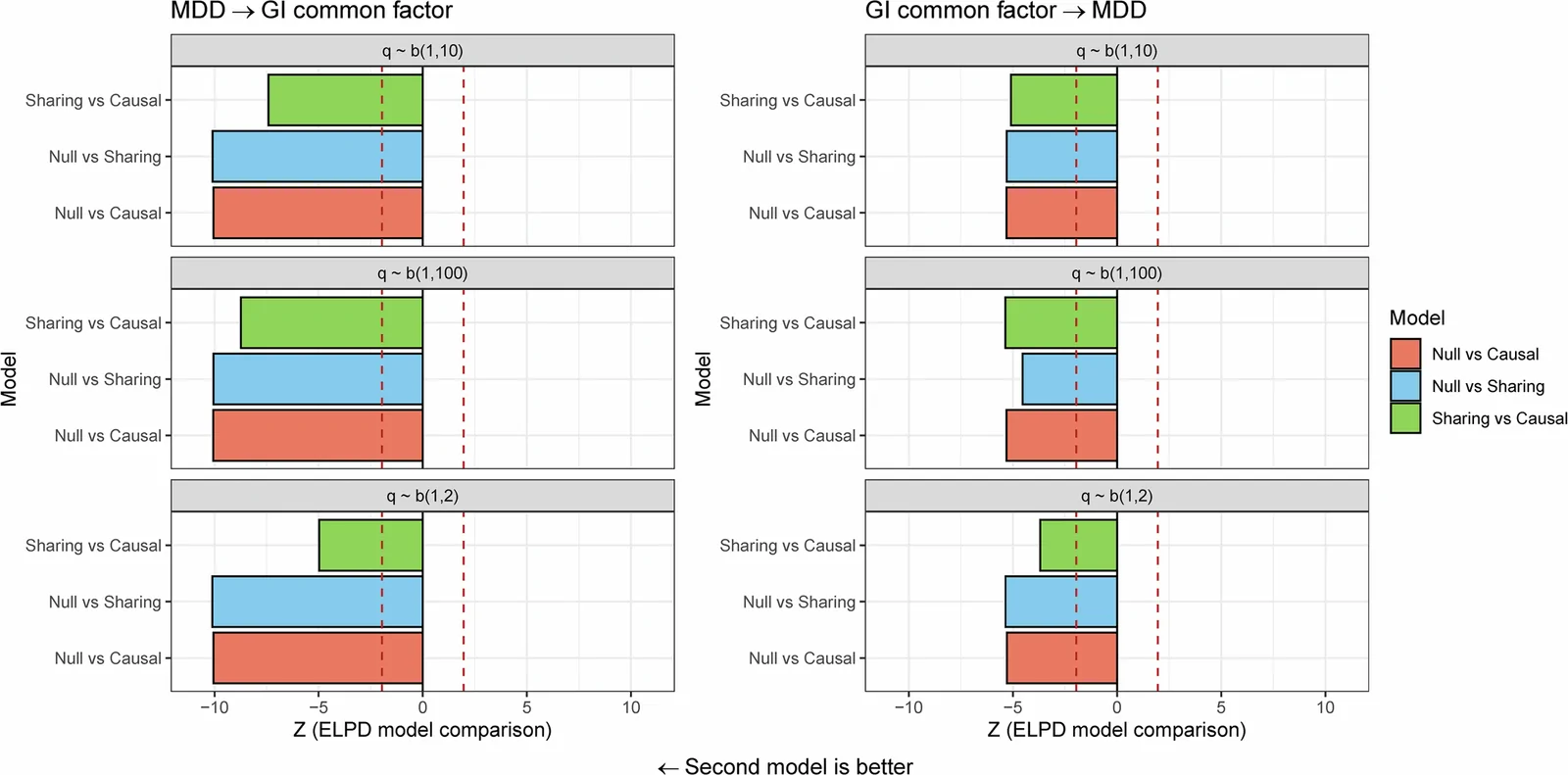
Exploring the relationship between major depressive disorder (MDD) and the gastrointestinal (GI) common factor using the CAUSE model. CAUSE models were constructed in both directions: MDD to GI common factor (left-hand side) and GI common factor to MDD (right-hand side). The *z* score of the change in expected log pointwise posterior density (ELPD/standard error of ELPD) is plotted between the two models in three configurations: null versus sharing, null versus causal and sharing versus causal. Three different β priors were implemented: q ~ β(1,10), q ~ β(1,2) and q ~ β(1,100). The red dotted lines denote nominal statistical significance (*P* < 0.05). Negative ELPD values imply the second listed model fits better. ELPD, expected log pointwise posterior density. Source data

We further explored MDD and the gastrointestinal cluster to elucidate whether this strong relationship arises due to genetic risk acting through the same pathway (horizontal pleiotropy) or acting through one trait that influences the other (vertical pleiotropy) using the latent causal variable (LCV)^34^ and causal analysis using summary effect (CAUSE)^35^ models. We found evidence of a bidirectional causal effect (vertical pleiotropy) between MDD genetic liability and the gastrointestinal cluster, with a slightly stronger effect in the direction from MDD to the gastrointestinal cluster (Extended Data Fig. 2 and ‘Estimating direct and shared genetic effects’ in Supplementary Results). However, due to high levels of correlated pleiotropy, CAUSE results may be inflated. This analysis should be interpreted cautiously, and further work is needed to determine the true extent of this relationship.

### Functional annotations of second-order factors

Next, we performed a competitive gene set analysis to identify biological pathways (Gene Ontology (GO) gene sets) and drug Anatomical Therapeutic Chemical (ATC) codes enriched in the heritability of each second-order factor using GSA-MiXeR^36^ (‘Gene-set analysis’ in Supplementary Results). We also conducted analyses of the univariate MDD GWAS for comparison (MDD only). Each second-order factor demonstrated enrichment for GO gene sets related to the central nervous system (CNS) that was not observed in the MDD-only analysis. Additionally, ATC drug codes associated with psychiatric treatment were found at Levels 3 and 4 of the ATC code hierarchy, supporting a psychosomatic component captured by the latent factors. The CVD and metabolic–MDD latent factors exhibited gene set enrichment related to cardiometabolic biological pathways. The CVD, metabolic and gastrointestinal–MDD factors showed enrichment for ATC codes related to their respective disease clusters. The propulsive drug–gene set, used to improve gut motility, was a mutually enriched code across the MDD-only, CVD, gastrointestinal and immune–MDD factors, supporting associations between physical disease comorbidities and MDD linked by the gut–brain axis. In addition, anxiolytics (drugs used to treat anxiety) were observed across these second-order factors but not in the MDD only. GSA-MiXeR does not produce formal *P* values, so these results were compared with pre-filtered MAGMA gene set analysis for a more conservative approach. Overall, few gene sets were observed in both analyses. MAGMA identified minimal associations, with most being larger gene sets and gene sets from the well-powered metabolic–MDD factor compared with those observed in GSA-MiXeR, highlighting the differences and limitations between the two approaches^36^.

**Fig. 5:**
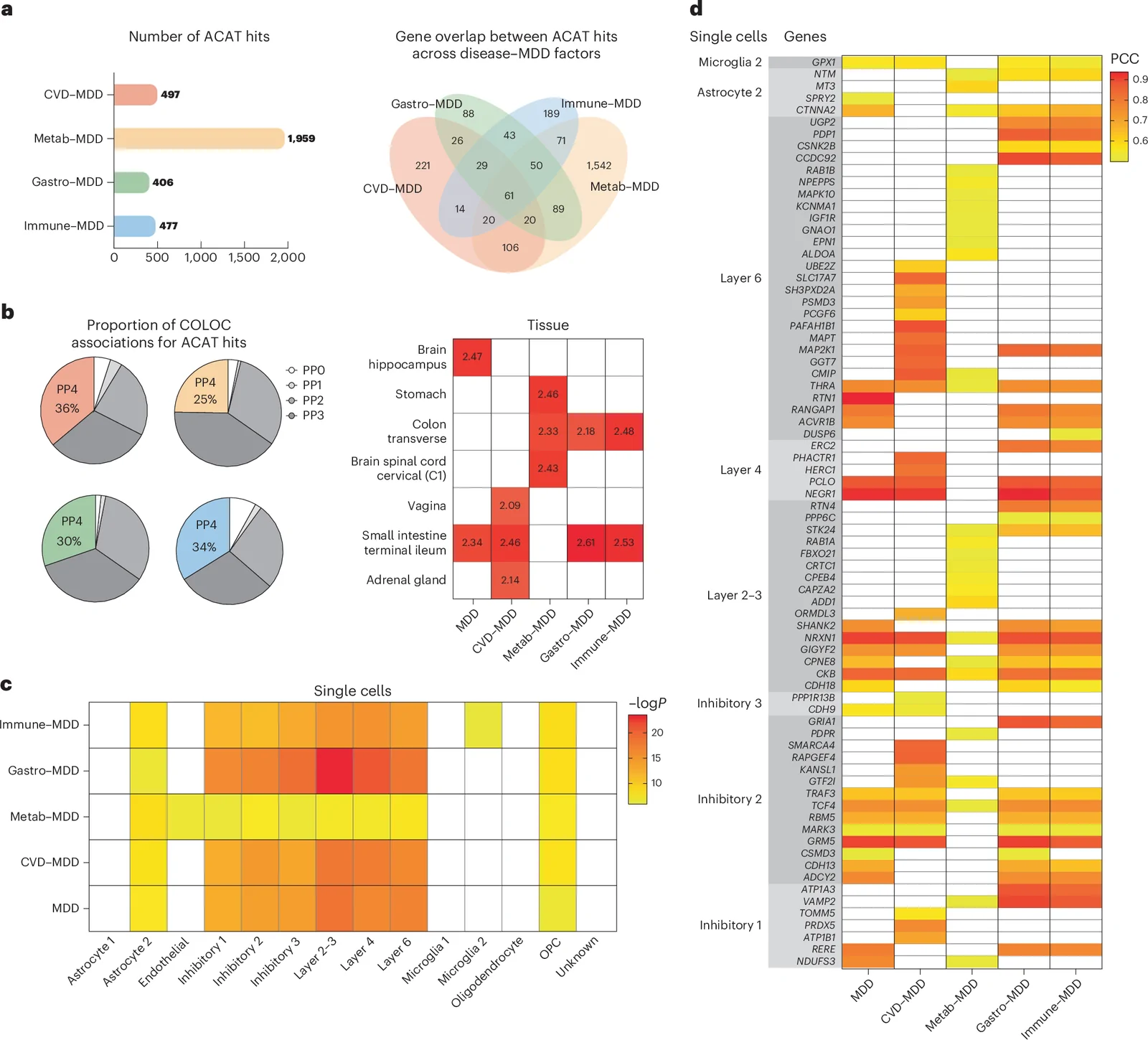
Functional annotations of second-order disease–MDD factors. **a**, The number of ACAT hits per tissue and their overlap across disease–MDD factors (tissue-level *P* values combined via ACAT, testing global null of no association across tissues; ACAT *P* < 0.05/number of genes tested per factor). **b**, The proportion of colocalized genes for ACAT hits per factor (PP4 > 0.80) and tissues with a high proportion of colocalized genes (coloc genes/number of genes), normalized to *z* scores to compare across factors (*z* > 1.96). **c**, gsMAP results using human brain cortex tissue to identify single-cell associations across disease–MDD factors and MDD only using the Cauchy combination test and corrected for multiple testing (*P* < 0.05/14). **d**, gsMAP results using human brain cortex tissue to identify cell type-specific gene associations with each factor and MDD only (PCC > 0.50; MAGMA *P* < 0.05/19,154). Cardiovascular cluster, red; metabolic cluster, yellow; gastrointestinal cluster, green; immune cluster, blue. OPC, oligodendrocyte precursor cell; PCC, Pearson correlation coefficient. Source data

We constructed a transcriptome-wide structural equation model to determine gene expression patterns across tissues (*n* = 45) and identify genes with expression associated with each second-order factor using genomic SEM and FUSION^37^ (Methods). We performed two additional analyses, including an aggregated Cauchy association test^38^ (ACAT) to meta-analyze *P* values and determine genes with a significant effect across tissues (ACAT hits), and colocalization to pinpoint variants with a high likelihood (PP4 > 0.80) to be associated with the disease–MDD factor and changes in gene expression, suggesting putative causal genes (COLOC genes). The ACAT analysis identified 2,569 unique significant genes across disease–MDD factors expressed in at least one tissue, with 2,274 not identified in MDD univariate analysis (Fig. 5a). We next examined the proportion of colocalized genes across tissues, normalized to *z* scores to compare across disease clusters. We observed a high proportion of colocalized genes (*z* > 1.96) in the gastrointestinal tract, including stomach, small intestine and colon tissue in disease–MDD factors and MDD only (Fig. 5b). Additionally, the adrenal gland had a high proportion of colocalization signal in the CVD–MDD factor, which may link to the hypothalamic–pituitary–adrenal axis, a possible shared driving mechanism of depression and CVD^39^.

Lastly, we conducted spatial transcriptomics analysis using gsMAP and single-cell data from the human brain cortex. This method integrates spatial transcriptomic data with GWAS summary statistics to map cell types to complex traits and identify cell type-specific gene associations. Inhibitory cell types, astrocytes, oligodendrocyte progenitor cells and layers 2, 3, 4 and 6 of the brain cortices showed significant signals across factors (*P* Cauchy < 0.05/14, corrected for the number of cell types; Fig. 5c). The immune–MDD factor displayed a distinct association with the immune cell type microglia, indicating potential immune mechanisms in the brain related to the shared liability of immune diseases and MDD. Figure 5d illustrates gene associations relevant to specific cell types and latent factors. Gene associations were identified in the disease–MDD factors that were not recognized in MDD alone. These findings may point to mechanisms specific to the shared genetic liability of physical disease and MDD, with cells in layer 6 exhibiting unique gene associations across various factors.

## Discussion

In this study, we sought to dissect and characterize pleiotropy in MDD and comorbid physical diseases. Our analysis revealed that three disease clusters explained an independent portion of the genetic component of MDD. Notably, the gastrointestinal disease cluster had the strongest association with MDD, accounting for a substantial impact from other disease clusters, influenced by functional gastrointestinal disorders and the gut–brain axis. Additionally, we highlighted critical pleiotropic components associated with disease–MDD latent factors, including loci, tissues, single cells and genes distinct from those associated with MDD alone. These elements may be pivotal genetic drivers of MDD–physical disease comorbidities.

In line with prior research^24^, we demonstrate a shared genetic basis underlying the gut–brain axis, MDD and gastrointestinal diseases. Our results expand on these findings, showing that this is likely due to psychopathological pathways related to functional gastrointestinal diseases that are associated with other disease systems. For example, a mechanistic hypothesis for CVD–MDD comorbidity is that it is mediated by immune–metabolic pathways^40^. Our shared-liability framework suggests that immune–metabolic genetic liability largely overlaps with the gastrointestinal cluster rather than contributing independent components. In other words, gut–brain mechanisms may act and partially overlap with immune–metabolic pathways. The gut–brain axis has exhibited connections with CVD^41^, diabetes^42^, metabolic syndrome^43^ and immune diseases^44^. We observed that this axis may influence the genetic relationship of these diseases with MDD.

The enteric nervous system (ENS) is a key component of the gut–brain relationship. The ENS and CNS share neurotransmission, signaling cascades, and structural features, which often result in CNS pathology emerging in enteric systems^45^. We observed that gastrointestinal–MDD shared genetic liability was associated with functional gastrointestinal disease, gene regulatory signals in digestive tissues and drug classes that enhance gut motility (for example, propulsives). Together with the identified neuronal gene sets, these findings suggest that neural mechanisms of gut function may contribute to this genetic overlap and point to ENS-related manifestations. We observed the dopaminergic system as an enriched neurotransmitter system, with particular emphasis on the D2 receptor in both MDD and gastrointestinal liability. This receptor has been shown to be a key communicator between the gut, brain and microbiota^46^, is associated with multiple gastrointestinal diseases^47^, and shows digestive benefits when targeted with medication^47^. For example, many gastrointestinal treatments (for example, domperidone^47^) are D2 receptor inhibitors or antagonists, for which we show upregulated expression in gastrointestinal tissues. Additionally, the ENS is tightly coupled with immune-related mechanisms^48^, which may contribute to the observed attenuation of MDD–immune associations. Further research should examine the CNS–ENS relationship in MDD, including immune responses and the manifestations of MDD–physical disease liability in neurotransmission, particularly dopaminergic pathways.

Current treatments for MDD are not effective for all patients, with physical comorbidities influencing treatment outcomes^49^. However, addressing pleiotropic components may have a broader impact on comorbidities^50^. Management of MDD comorbidities has demonstrated synergistic benefits; for example, antidepressants have positively influenced physical disease outcomes^29^, such as glycemic control in diabetics^51^. Conversely, certain physical disease treatments enhance the effects of antidepressants (for example, anti-inflammatory medications^52^ and statins^53^). Concerning functional gastrointestinal disorders, both psychotropic and psychological interventions have proven effective in alleviating gastrointestinal symptoms^54,55^. Our findings accentuate the crucial psychopathological connections related to the gut–brain axis, presenting it as a key mechanism in physical disease comorbidities and a likely pathway affecting treatment outcomes. Considering the bidirectional nature of these conditions, treatment may require a multidisciplinary approach that encompasses addressing these pathways alongside managing physical symptoms. Future research should investigate disease causality, the impact of the gut microbiome and the role of environmental factors, such as diet, to improve treatment outcomes.

The findings of this study should be interpreted in light of the following limitations and considerations. First, our study was limited to individuals of European ancestry due to data availability. Second, the current GWAS sample sizes did not allow the inclusion of other physical disease associations (for example, immune disorders such as psoriasis vulgaris, systemic lupus erythematosus^56^). Third, broad phenotypic definitions used in the MDD GWAS may increase genetic heterogeneity and dilute disorder-specific signals and MDD subtypes^57^ (for example, inflammatory depression), while also inflating trait overlap with proximal or related symptoms (for example, functional gastrointestinal symptoms). Consequently, the physical disease–MDD associations should be interpreted pertaining to the cohort’s general ascertainment rather than a strict, clinically uniform MDD phenotype. Fourth, the results are restricted to common genetic variants and assume a uniform distribution of effect sizes.

It is important to consider the differences in the power of disease cluster traits, which are influenced by effective sample size and SNP heritability. In genomic SEM, power impacts the precision of LDSC inputs, factor measurements and statistical significance of path coefficients. We observed model instability at a lower trait *z*-score cutoff threshold (*h*^2^_SNP_ *z* > 4; ‘Disease–MDD latent factor sensitivity analysis (SNP-based heritability *z* > 4)’ in Supplementary Results), and notably, the immune cluster became insignificant in the joint model, a change that may partly reflect its lower power. Furthermore, differences in the number of shared loci or enriched functional annotations should not be interpreted as evidence for stronger biological overlap with MDD, as these patterns may reflect variations in GWAS power. For example, the metabolic–MDD cluster showed strong enrichment in gene set analysis and identified the most loci; however, comparatively, the metabolic cluster has the most power. Similarly, differences in power with MDD led to more pronounced effects on the second-order factors, despite equivalent loadings. For instance, the metabolic–MDD factor showed a stronger metabolic association, causing the nervous system and psychiatric enrichment to appear relatively diluted.

Lastly, the immune cluster was not independently associated with MDD in the joint model. This does not indicate that immune mechanisms are unrelated to MDD; rather, this cluster’s association with MDD is shared with other factors, particularly the gastrointestinal factor, which may reflect the strong link between gastrointestinal disorders and immune mechanisms^58^. However, we note several limitations of the immune cluster that may influence these findings. This cluster had the smallest shared variance among the individual traits, as evidenced by its weaker genetic correlations and factor loadings, consistent with a less uniform shared signal. A potential cause is the heterogeneous nature of immune disorders, with various subtypes, immune responses and effects across different tissues and organs. Cross-disorder genetic analyses have revealed distinct clusters of genetic associations among immune-mediated diseases^59^ as well as psychiatric factors associated with immune subtypes to different degrees^60^. Additionally, the major histocompatibility complex (MHC) region was removed from genomic SEM and LDSC due to its complex LD structure, which may also influence the results for the immune cluster, given that human leukocyte antigen type is often the strongest common genetic risk factor for immune-driven disorders. With larger sample sizes for immune traits, future work should examine the various immune subtypes and the influence of the MHC region on physical disease–MDD comorbidities.

In conclusion, our findings highlight the shared genetic architecture between MDD and physical disease clusters. These results emphasize the complex interactions among physical disease systems and their interplay with MDD, suggesting genetic factors that may influence the management of comorbid physical diseases.

## Methods

### Ethics

No individual-level data were used. This study only used summary-level data. Ethical approval was obtained in the original studies (for further details on each GWAS, see ref. ^21^ and Supplementary Table 1).

### Data selection and processing

The MDD summary statistics from ref. ^21^ (excluding 23andMe) were used for this analysis. To identify traits for inclusion in each disease group (cardiovascular, metabolic, gastrointestinal and immune), we searched the published literature for disorders and traits commonly comorbid with MDD and related to each group. GWAS summary statistics of potential traits were downloaded mostly from data repositories (GWAS Catalog and the National Bioscience Database Center Human Database; for full details, see Supplementary Table 1 and ‘Data selection’ in Supplementary Results). Only data from the largest European-ancestry studies were considered due to the limited availability of well-powered GWAS of other ancestries. Summary statistics were processed using the ‘munge’ function within the genomic SEM R package (v0.0.5)^27^ with default parameters and the 1000 Genomes European linkage disequilibrium (LD) reference panel. SNPs were filtered using an INFO filter of 0.90 and a minor allele frequency filter of 0.01 if this information was available.

### LD score regression

To determine which traits have significant genetic overlap with MDD, we applied LDSC^61^ using the ‘ldsc’ function in the genomic SEM R package with default settings. LDSC estimates genome-wide bivariate correlations and accounts for sample overlap. Due to its complex LD structure, the MHC region was excluded from the analyses. SNP-based heritability for each trait was estimated using LDSC, with traits retained based on two SNP-based heritability *z*-score thresholds, *z* > 4 and *z* > 7. To identify MDD-related latent factors, traits that passed the threshold and were significantly correlated with MDD after correction for multiple testing (genetic covariance *P* < 0.05 per number of traits above the threshold) were used for latent factor analysis.

We retained a representative set of traits for each disease group (Table 1). All groups contained four traits. The cardiovascular group included atrial fibrillation, coronary artery disease, heart failure and stroke. The metabolic group comprised high-density lipoprotein cholesterol, metabolic syndrome, type 2 diabetes and triglycerides. The gastrointestinal group included gastroesophageal reflux disease, gallstone disease, irritable bowel syndrome and peptic ulcer disease. The immune group comprised atopic dermatitis, asthma, inflammatory bowel disease and multiple sclerosis.

### Latent factor models

In all described models, parameters were estimated using diagonally weighted least squares, and two indices were used to evaluate model fit. These indices included CFI and SRMR, with thresholds of CFI > 0.90 and SRMR < 0.10 for acceptable fit. Additionally, in instances of a Heywood case, constraints were applied to amend the discrepancy, such as fixing a negative variance to greater than zero.

#### Common factor models

We fitted factor models for each disease group separately to assess whether a factor could capture the shared variance across the identified traits and estimate the overall relationship between MDD and the disease clusters. We used the ‘usermodel’ function in genomic SEM^27^ to estimate the models using unit variance identification. For each disease group, individual traits loaded onto a single common latent factor. MDD was then regressed on the latent factor to estimate the genetic association between the disease factors and MDD.

#### Four-factor multiple regression model

We combined the previously identified models into a four-factor model. This model was fitted in genomic SEM to determine the independent associations of each disease latent factor with the heritable component of MDD. This model also enabled estimation of the residual variance of MDD unexplained by the four physical disease groups. MDD was simultaneously regressed onto each disease group’s latent factor, with all factors correlating with each other. Unit factor identification was used for model identification. Traits that showed high residual correlations across other disease clusters (>0.10) were allowed to load onto multiple factors if this improved model fit, as assessed using a *χ*^2^ test and CFI and SRMR estimates.

#### Exploratory factor analysis

We performed an exploratory factor analysis to compare the number of factors and loading patterns suggested by the data without imposing the latent structure specified in the confirmatory model. We used the LDSC covariance matrix and the ‘fa’ function in the ‘psych’ R package^62^, with oblique rotation (oblimin) and maximum likelihood (ml) settings. Factors with eigenvalues greater than one were extracted, and a loading cutoff of 0.30 was used to define strong loadings.

#### Directed acyclic graphs

To examine potential mediators between physical disease clusters and MDD, we constructed mediation models in genomic SEM. These models were based on hypothesized relationships identified in previous analyses. We used thresholds of CFI > 0.90 and SRMR < 0.10 for acceptable fit with models estimated using diagonally weighted least squares.

#### Multiple regression of individual traits

We specified a multiple regression model in genomic SEM by regressing MDD onto the traits that comprised the disease factors. We used this approach to identify specific traits driving the relationship between the disease factor and MDD, highlight unique associations with MDD not captured by the factor and compare the MDD variance explained by individual traits versus the latent factor. We included traits encompassing a latent factor with a significant independent association in the multiple regression model.

### Multivariate GWAS

#### Second-order latent factor models

We performed a multivariate GWAS within genomic SEM for each disease group and MDD. We developed higher-order factor models using a second-order latent factor loaded onto the disease group latent factors and MDD, with equality constraints fixed on these loadings to identify the model. This second-order factor captures the shared variance between the disease factors and MDD, enabling further characterization of their genetic overlap and identification of variants broadly affecting disease groups and MDD. This model hypothesizes that a common genetic factor mediates the relationship between MDD and the disease group and that this relationship is mediated equally. We used the ‘userGWAS’ function with a diagonally weighted least squares estimator to conduct the multivariate GWAS. The model was identified using unit loading identification by fixing the factor loading of the trait with the largest loading from the common factor model to 1.

#### Independent loci and *Q*_SNP_ heterogeneity

Two models were produced to filter for SNP heterogeneity effects measured using *Q*_SNP_. *Q*_SNP_ is a *χ*^2^-distributed test statistic, with higher values indicating that a factor does not entirely explain a SNP’s effect. In the first model, the SNP was loaded onto the second-order latent factor, that is, the SNP acting through a common pathway. In the second model, the SNP was loaded onto MDD and the disease factor, representing the SNP acting through independent pathways. The *χ*^2^ values of the common and independent pathways were then compared to derive the *Q*_SNP_ heterogeneity metric. SNPs were removed if their effects were consistent with the independent pathway or if they were in LD with a significant *Q*_SNP_. We used the PLINK v1.90 ‘clump’ function to identify SNPs in LD with *Q*_SNP_ (*P*_1_ = 5 × 10^−8^; *P*_2_ = 5 × 10^−4^; *R*^2^ = 0.10; kb = 1,000). This approach ensures that any identified loci are significant for the shared factor rather than for the disease group factor and MDD independently.

We uploaded the summary statistics of each multivariate GWAS to FUMA (v1.5.2)^63^. Independent significant loci were defined using the PLINK clumping procedure in FUMA with default settings (*r*^2^ > 0.6, LD block distance > 250 kb) and the 1000 Genomes Phase 3 European reference panel. The ‘Open Targets Platform’^64^ was used to identify previously unidentified loci using the ‘Google BigQuery’ platform and the ‘Open Targets Genetics’ datasets ‘variants’ and ‘variants-disease’. A locus was classified as previously unidentified if there were no disease-associated lead or tag variants within the locus for European ancestry in the Open Targets Platform and it was not observed in the original GWASs. This was tested for both MDD (loci previously unidentified for MDD) and the traits encompassing the disease groups (loci previously unidentified for the diseases). Loci unique to each disease–MDD factor were determined using the ‘bedtools^65^ intersect’ function. An independent locus with no overlap with any locus in the other second-order factors was categorized as unique.

### Characterization of physical disease–MDD associations

#### PheWASs

We conducted PheWASs to characterize the disease–MDD loci. PheWASs investigate associations between a wide range of phenotypes and a variant or set of variants. Using the previously described ‘Open Targets Platform’^64^ datasets, known trait associations were mapped to the identified loci. Traits were categorized into their respective groups, and the number of associations was counted. Unique genome-wide significant variant–trait associations of European ancestry were considered. This was performed for each disease–MDD factor, and associations were partitioned into loci previously unidentified for disease traits, loci previously unidentified for MDD, and loci unique to the second-order factor.

#### Social and behavioral traits

We conducted multiple regression using MDD and the physical disease latent factors to examine associations with social and behavioral traits. Common social and behavioral traits associated with MDD were selected (Supplementary Table 24). We performed LDSC in the genomic SEM R package on these traits, physical disease common factors and MDD using default parameters. Next, in genomic SEM, we compared two models for each social and behavioral trait. First, MDD was regressed separately on the trait and each factor to estimate baseline regression coefficients (MDD ~ Trait; MDD ~ Factor). Second, multiple regression models were fitted in which MDD was regressed on both the social or behavioral trait and the physical disease factor (MDD ~ Trait + Factor).

### Gastrointestinal factor follow-up analysis

#### GORD subtypes

To investigate the association between the gastrointestinal cluster and functional subtypes, we obtained GORD variants listed in ref. ^33^ and summary statistics for the gastrointestinal–MDD latent factor. Ref. ^33^ derived GORD subtypes from variants that were depression-driven (functional-GORD associated) and BMI-driven (nonfunctional-GORD associated). Using a Wilcoxon test, we compared the absolute effect sizes of these variants from the gastrointestinal–MDD GWAS to explore their relationship with these gastrointestinal subtypes. We also implemented LDSC in the genomic SEM R package to estimate genetic correlations with MDD and GORD subtypes. LDSC was performed with MDD and the other gastrointestinal traits using default settings.

#### LCV

We used the LCV model^34^ to estimate evidence of a causal relationship in either direction between the gastrointestinal common factor and MDD. This analysis models the bivariate distribution of genome-wide effects from both GWASs (MDD and gastrointestinal common factor). Bias arising from sample overlap is mitigated in the LCV framework using the LDSC intercept. In our LCV model, we extracted ~1 million common HapMap3 non-MHC variants from munged summary statistics for both traits, setting MDD as trait one and the gastrointestinal common factor as trait two. We used the recommended threshold of significantly nonzero |GCP| > 0.6, where GCP denotes the genetic causality proportion, to define partial genetic causality in either direction^34^.

#### CAUSE

We used the CAUSE model^35^ to estimate model parameters of correlated horizontal pleiotropy and direct or vertical pleiotropic effects between the gastrointestinal common factor and MDD. The CAUSE model was implemented using the CAUSE R package (v1.2.0), with sample overlap directly accounted for empirically during estimation of the correlation between variants. Models were built in both directions (MDD → gastrointestinal common factor; gastrointestinal common factor → MDD). Approximately 1 million random variants from the GWAS were used to estimate nuance parameters in the model, followed by specifically using a set of clumped variants that were at least nominally associated with the exposure to perform model comparison (*P* < 0.001, *r*^2^ < 0.01, 1000 Genomes Project Phase 3 European reference panel), in line with recommendations for the CAUSE method. Three *β* prior distributions were tested with asymmetric hyperparameters (necessary for the model to be identifiable) for the *q* parameter, with *q* the proportion of variants that act through correlated pleiotropy—*q* ~ *β*(1,2), *q* ~ *β*(1,10) and *q* ~ *β*(1,100)^35^.

### Biological annotations

#### Gene set enrichment

GSA-MiXeR^36^ (v2.1.1), an analytical tool for exploratory gene set enrichment, was used for each second-order factor and MDD alone. This analysis models gene set heritability enrichment and enables the quantification of partitioned heritability and fold enrichment for complex traits. GO gene sets and the 1000 Genomes Project Phase 3 European reference panel provided by GSA-MiXeR were downloaded from https://github.com/precimed/gsa-mixer. ATC code drug–gene sets were obtained from DrugBank^66^. Gene sets with Akaike information criterion > 0 and enrichment scores >1 were explored, with analyses performed using default parameters recommended by the software. Additionally, MAGMA gene set analysis^67^ was performed alongside GSA-MiXeR as a conservative comparison. While GSA-MiXeR alone has been shown to be stable in gene set rankings, this method does not produce formal *P* values; therefore, it relies on MAGMA as an additional step to prefilter gene sets using Bonferroni multiple-testing correction (0.05 per number of gene sets). We report both exploratory (GSA-MiXeR without prefiltering) and conservative (GSA-MiXeR with MAGMA) approaches.

#### Gene expression of second-order factors

Transcriptome-wide SEM (T-SEM) was performed to determine tissue-specific imputed gene expression patterns for second-order latent factor models. First, univariate transcriptome-wide association studies were implemented for each trait using the FUSION^37^ software to impute genetically regulated gene expression using default settings. We downloaded 45 European-specific tissue weights derived from the Genotype-Tissue Expression (GTEx) Program (v8)^68^ from the FUSION website, which excluded nonnatural tissues (cell cultures), tissues with <100 samples and an expression outlier (testis tissue^69^). Second, the T-SEM function in the genomic SEM R package (v0.0.5) was implemented for this analysis. The previously determined LDSC output and second-order factor models from multivariate GWAS were used. Like *Q*_SNP_, a *Q*_GENE_ is a *χ*^2^-distributed test statistic calculated to filter out genes not operating through the common pathway model, removing genes with a *P* < 0.05 per number of genes per tissue. ACAT R package (v0.91)^38^ was used to meta-analyze *P* values and identify tissues with significant effects across tissues (ACAT hits; *P* < 0.05 per number of genes tested per factor). Colocalization was performed for each second-order factor GWAS using the FUSION software and the same tissue weights as in the previous analysis. The coloc results from this analysis were integrated with the T-SEM results to determine genes with a high probability (PP4 > 0.80) of being associated with changes in gene expression and the disease–MDD factor, indicating that a gene is putatively causal. *Z* scores for colocalization gene proportions (that is, COLOC genes/total genes in tissue) were calculated to normalize and compare colocalization signals across factors.

#### gsMAP

Genetically informed spatial mapping of cells for complex traits (gsMAP)^70^ was conducted by integrating spatial transcriptomic data with GWAS summary statistics to identify cell types and single-cell-specific genes associated with each second-order factor and MDD. We used spatial transcriptomic data from the human brain frontal cortex obtained using the CosMx Spatial Molecular Imager profiling over 6,000 genes at single-cell and subcellular resolution from formalin-fixed paraffin-embedded human brain tissue^71,72^. Cell types were determined using the Cauchy combination results, highlighting specific cell types for each factor and MDD (*P* < 0.05/14). We prioritized cell type-specific genes based on gsMAP Pearson correlation coefficient > 0.50 and MAGMA *P* values using gene mapping in FUMA^63^ (window = 10 kb; *P* < 0.05/19,154). Pearson correlation coefficient summarizes the relationship between spatial gene expression in cell types and their genetic enrichment. MAGMA provides an additional layer of evidence supporting the gene as highly relevant to the disease.

### Reporting summary

Further information on research design is available in the Nature Portfolio Reporting Summary linked to this article.

## Online content

Any methods, additional references, Nature Portfolio reporting summaries, source data, extended data, supplementary information, acknowledgements, peer review information; details of author contributions and competing interests; and statements of data and code availability are available at https://doi.org/10.1038/s41588-026-02735-3.

## Supplementary information

Supplementary Information Supplementary Results and Supplementary Figs. 1–11.

Reporting Summary

Peer Review File

Supplementary Tables Supplementary Tables 1–56.

## Source data

Source Data Fig. 2 Source data.

Source Data Fig. 3 Source data.

Source Data Fig. 4 Source data.

Source Data Fig. 5 Source data.

Source Data Extended Data Fig. 1 Source data.

Source Data Extended Data Fig. 2 Source data.

## Data Availability

The MDD GWAS summary statistics (excluding 23andMe) were downloaded from https://ipsych.dk/en/research/downloads/. See Supplementary Table 1 for download links to publicly available physical disease GWAS summary statistics. MS GWAS summary statistics were obtained from https://imsgc.net/ upon request. The Open Targets Platform datasets were accessed via Google BigQuery (https://platform-docs.opentargets.org/data-access/google-bigquery). GO gene sets were downloaded from https://github.com/precimed/gsa-mixer, and ATC codes gene sets from https://go.drugbank.com/. EUR GTEx v8 tissue weights are available from the FUSION website (http://gusevlab.org/projects/fusion/). The single-cell spatial dataset of the formalin-fixed paraffin-embedded human frontal cortex was obtained from NanoString (https://nanostring.com/products/cosmx-spatial-molecular-imager/ffpe-dataset/human-frontal-cortex-ffpe-dataset/). GWAS summary statistics for second-order factors are available at https://doi.org/10.6084/m9.figshare.32916761. Source data are provided with this paper.

## Code availability

Analyses were performed using the following publicly available software: PLINK (v1.90; https://www.cog-genomics.org/plink/1.9/), bedtools (v2.31.1; https://bedtools.readthedocs.io/en/latest/), FUMA (v1.5.2; http://fuma.ctglab.nl/), LCV (https://github.com/lukejoconnor/LCV), GSA-MiXeR (v2.1.1; https://github.com/precimed/gsa-mixer), MAGMA (v1.10; https://cncr.nl/research/magma/), TWAS FUSION (http://gusevlab.org/projects/fusion/), gsMAP (v1.71.2; https://github.com/JianYang-Lab/gsMap). RStudio (v4.3.1) was used. R packages included the genomic SEM R package (v0.0.5; https://github.com/GenomicSEM/GenomicSEM), the psych R package (v2.4.6.26; https://cran.r-project.org/web/packages/psych/index.html), the Aggregated Cauchy association test R package (v0.91; https://github.com/yaowuliu/ACAT), CAUSE R package (v1.2.0; https://github.com/jean997/cause). Code used in this analysis is available at https://github.com/damian-wo/MDD-pleio.git and https://doi.org/10.5281/zenodo.21711189 (ref. ^73^).
