# Supplementary material for: Dissecting pleiotropy between major depressive disorder and physical disease comorbidities
Source: Nat Genet. 2026 Aug 26;58(9):2201–10. doi: 10.1038/s41588-026-02735-3 (PMC13553304; doi:10.1038/s41588-026-02735-3)
Supplement: Supplementary file 2 [file 41588_2026_2735_MOESM2_ESM.pdf]

nature portfolio

Corresponding author(s): Damian Woodward

Last updated by author(s): Jul 27, 2026

# Reporting Summary

Nature Portfolio wishes to improve the reproducibility of the work that we publish. This form provides structure for consistency and transparency in reporting. For further information on Nature Portfolio policies, see our Editorial Policies and the Editorial Policy Checklist.

## Statistics

For all statistical analyses, confirm that the following items are present in the figure legend, table legend, main text, or Methods section.

| n/a | Confirmed | |
|---|---|---|
| ☐ | ☒ | The exact sample size ($n$) for each experimental group/condition, given as a discrete number and unit of measurement |
| ☒ | ☐ | A statement on whether measurements were taken from distinct samples or whether the same sample was measured repeatedly |
| ☐ | ☒ | The statistical test(s) used AND whether they are one- or two-sided<br>*Only common tests should be described solely by name; describe more complex techniques in the Methods section.* |
| ☒ | ☐ | A description of all covariates tested |
| ☐ | ☒ | A description of any assumptions or corrections, such as tests of normality and adjustment for multiple comparisons |
| ☐ | ☒ | A full description of the statistical parameters including central tendency (e.g. means) or other basic estimates (e.g. regression coefficient) AND variation (e.g. standard deviation) or associated estimates of uncertainty (e.g. confidence intervals) |
| ☐ | ☒ | For null hypothesis testing, the test statistic (e.g. $F$, $t$, $r$) with confidence intervals, effect sizes, degrees of freedom and $P$ value noted<br>*Give P values as exact values whenever suitable.* |
| ☐ | ☒ | For Bayesian analysis, information on the choice of priors and Markov chain Monte Carlo settings |
| ☒ | ☐ | For hierarchical and complex designs, identification of the appropriate level for tests and full reporting of outcomes |
| ☐ | ☒ | Estimates of effect sizes (e.g. Cohen's $d$, Pearson's $r$), indicating how they were calculated |

*Our web collection on statistics for biologists contains articles on many of the points above.*

## Software and code

Policy information about availability of computer code

| | |
|---|---|
| Data collection | The Open Targets Platform datasets were accessed via Google BigQuery (https://platform-docs.opentargets.org/data-access/google-bigquery). |
| Data analysis | Analyses were performed using publicly available software: PLINK v1.90 (https://www.cog-genomics.org/plink/1.9/), bedtools v2.31.1 (https://bedtools.readthedocs.io/en/latest/), FUMA v1.5.2 (http://fuma.ctglab.nl/), LCV (https://github.com/lukejoconnor/LCV), GSA-MiXeR v2.1.1 (https://github.com/precimed/gsa-mixer), MAGMA v1.10 (https://cncr.nl/research/magma/), TWAS FUSION (http://gusevlab.org/projects/fusion/), gsMAP v1.71.2 (https://github.com/JianYang-Lab/gsMap).<br><br>RStudio version 4.3.1 was used. R packages included Genomic SEM R package v0.0.5 (https://github.com/GenomicSEM/GenomicSEM), psych R package v2.4.6.26 (https://cran.r-project.org/web/packages/psych/index.html), Aggregated Cauchy association test R package v0.91 (https://github.com/yaowuliu/ACAT), CAUSE R package v1.2.0 (https://github.com/jean997/cause). Code used in this analysis is available at https://github.com/damian-wo/MDD-pleio.git and https://doi.org/10.5281/zenodo.21711189. |

For manuscripts utilizing custom algorithms or software that are central to the research but not yet described in published literature, software must be made available to editors and reviewers. We strongly encourage code deposition in a community repository (e.g. GitHub). See the Nature Portfolio guidelines for submitting code & software for further information.

## Data

Policy information about availability of data

All manuscripts must include a data availability statement. This statement should provide the following information, where applicable:

- Accession codes, unique identifiers, or web links for publicly available datasets
- A description of any restrictions on data availability
- For clinical datasets or third party data, please ensure that the statement adheres to our policy

The MDD GWAS summary statistics (excluding 23andMe) were downloaded from https://ipsych.dk/en/research/downloads/. See Supplementary Table 1 for download links to publicly available physical disease GWAS summary statistics. MS was obtained from https://imsgc.net/ upon request. GO gene sets were downloaded from https://github.com/precimed/gsa-mixer, and ATC codes gene sets from https://go.drugbank.com/. EUR GTEx v8 tissue weights are available from the FUSION website (http://gusevlab.org/projects/fusion/). The single-cell spatial dataset of the formalin-fixed paraffin-embedded Human Frontal Cortex was obtained from nanoString (https://nanostring.com/products/cosmx-spatial-molecular-imager/ffpe-dataset/human-frontal-cortex-ffpe-dataset/). GWAS summary statistics for second-order factors are available at https://doi.org/10.6084/m9.figshare.32916761.

## Research involving human participants, their data, or biological material

Policy information about studies with human participants or human data. See also policy information about sex, gender (identity/presentation), and sexual orientation and race, ethnicity and racism.

| | |
|---|---|
| Reporting on sex and gender | See the original GWAS listed in Supplementary Table 1 for full reporting of sex and gender for their respective cohorts. |
| Reporting on race, ethnicity, or other socially relevant groupings | All analyses used summary-level GWAS data from participants of European ancestry. The exclusion of other groups was due to data availability. |
| Population characteristics | The population of the GWAS used were of European ancestry. See Supplementary Table 1 for a list of publications for more detailed information. |
| Recruitment | See Als et al. 2023 Nature Medicine and Supplementary Table 1 for publications listed. |
| Ethics oversight | No individual level data was used. This project only used summary-level data. Ethical approval was obtained in the original publications, see Als et al. 2023 Nature Medicine and Supplementary Table 1 for further details on each GWAS. |

Note that full information on the approval of the study protocol must also be provided in the manuscript.

# Field-specific reporting

Please select the one below that is the best fit for your research. If you are not sure, read the appropriate sections before making your selection.

☒ Life sciences ☐ Behavioural & social sciences ☐ Ecological, evolutionary & environmental sciences

For a reference copy of the document with all sections, see nature.com/documents/nr-reporting-summary-flat.pdf

# Life sciences study design

All studies must disclose on these points even when the disclosure is negative.

| | |
|---|---|
| Sample size | Sample sizes for each GWAS are listed in Table 1. Traits were removed if they did not meet required power thresholds as measured using LDSC (h2 SNP Z > 7). We selected disease traits relating to each physical disease cluster and had a significant genetic correlation with MDD. |
| Data exclusions | Only GWAS of European populations were used to ensure LD pattern consistency, high sample size and robust power. |
| Replication | This project used the largest GWAS available. There were no sufficiently large GWAS for replication analysis. |
| Randomization | No experimental groups were employed in this project. |
| Blinding | No group allocation was conducted. |

# Reporting for specific materials, systems and methods

We require information from authors about some types of materials, experimental systems and methods used in many studies. Here, indicate whether each material, system or method listed is relevant to your study. If you are not sure if a list item applies to your research, read the appropriate section before selecting a response.

## Materials & experimental systems

| n/a | Involved in the study |
| --- | --- |
| ☒ | ☐ Antibodies |
| ☒ | ☐ Eukaryotic cell lines |
| ☒ | ☐ Palaeontology and archaeology |
| ☒ | ☐ Animals and other organisms |
| ☒ | ☐ Clinical data |
| ☒ | ☐ Dual use research of concern |
| ☒ | ☐ Plants |

## Methods

| n/a | Involved in the study |
| --- | --- |
| ☒ | ☐ ChIP-seq |
| ☒ | ☐ Flow cytometry |
| ☒ | ☐ MRI-based neuroimaging |

# Plants

| | |
| --- | --- |
| Seed stocks | N/A |
| Novel plant genotypes | N/A |
| Authentication | N/A |
