# Supplementary material for: Dissecting pleiotropy between major depressive disorder and physical disease comorbidities
Source: Nat Genet. 2026 Aug 26;58(9):2201–10. doi: 10.1038/s41588-026-02735-3 (PMC13553304; doi:10.1038/s41588-026-02735-3)
Supplement: Supplementary file 3 [file 41588_2026_2735_MOESM3_ESM.pdf]

nature portfolio

Peer Review File

Dissecting Pleiotropy Between Major Depressive Disorder and Physical Disease Comorbidities

Corresponding Author: Mr Damian Woodward

Version 0:

Decision Letter:

18th November 2025

Dear Damian,

Your Letter "Dissecting Pleiotropy Between Major Depressive Disorder and Physical Disease Comorbidities" has been seen by three referees. You will see from their comments below that, while they find your work of interest, they have raised several relevant points. We are interested in the possibility of publishing your study in Nature Genetics, but we would like to consider your response to these points in the form of a revised manuscript before we make a final decision on publication.

To guide the scope of the revisions, the editors discuss the referee reports in detail within the team, including with the chief editor, with a view to identifying key priorities that should be addressed in revision, and sometimes overruling referee requests that are deemed beyond the scope of the current study. In this case, we ask that you address all technical points related to the genetic analyses and their interpretation (particularly points related to selection of traits and datasets and how these choices influence the resulting factor model) and extend the analyses where feasible as requested by the reviewers. We hope you will find this prioritized set of referee points to be useful when revising your study. Please do not hesitate to get in touch if you would like to discuss these issues further.

We therefore invite you to revise your manuscript taking into account all reviewer and editor comments. Please highlight all changes in the manuscript text file. At this stage, we will need you to upload a copy of the manuscript in MS Word .docx or similar editable format.

We are committed to providing a fair and constructive peer-review process. Do not hesitate to contact us if there are specific requests from the reviewers that you believe are technically impossible or unlikely to yield a meaningful outcome.

When revising your manuscript:

*1) Include a “Response to referees” document detailing, point-by-point, how you addressed each referee comment. If no action was taken to address a point, you must provide a compelling argument. This response will be sent back to the referees along with the revised manuscript.

*2) If you have not done so already, please begin to revise your manuscript so that it conforms to our Letter format instructions, available
here.
Refer also to any guidelines provided in this letter.

*3) Include a revised version of your Reporting Summary: https://www.nature.com/documents/nr-reporting-summary.pdf
It will be available to referees (and, potentially, statisticians) to aid in their evaluation if the manuscript goes back for peer review.
A revised checklist is essential for re-review of the paper.

Please be aware of our guidelines on digital image standards.

EXTENDED DATA FIGURES

When re-submitting your manuscript, please ensure that any supplementary figures and tables that are crucial to the

manuscript’s conclusions are converted into Extended Data figures and tables to increase visibility of these data. Extended Data figures and tables are online-only (present in the online PDF and full-text HTML versions of the paper), peer-reviewed display items that provide essential background to the article but are not included in the main article due to space constraints. A maximum of ten Extended Data display items (figures and tables) is permitted.

Please use the link below to submit your revised manuscript and related files:

Link Redacted

Note: This URL links to your confidential home page and associated information about manuscripts you may have submitted, or that you are reviewing for us. If you wish to forward this email to co-authors, please delete the link to your homepage.

We hope to receive your revised manuscript within 8-12 weeks. If you cannot send it within this time, please let us know.

Please do not hesitate to contact me if you have any questions or would like to discuss these revisions further.

Nature Genetics is committed to improving transparency in authorship. As part of our efforts in this direction, we are now requesting that all authors identified as ‘corresponding author’ on published papers create and link their Open Researcher and Contributor Identifier (ORCID) with their account on the Manuscript Tracking System (MTS), prior to acceptance. ORCID helps the scientific community achieve unambiguous attribution of all scholarly contributions. You can create and link your ORCID from the home page of the MTS by clicking on ‘Modify my Springer Nature account’. For more information, please visit www.springernature.com/orcid.

We look forward to seeing the revised manuscript and thank you for the opportunity to review your work.

Sincerely,
Kyle

Kyle Vogan, PhD
Senior Editor
Nature Genetics
https://orcid.org/0000-0001-9565-9665

Referee expertise:

Referee #1: Genetics, psychiatric disorders, pleiotropy

Referee #2: Genetics, psychiatric disorders, cardiometabolic disorders

Referee #3: Genetics, mood disorders, depression

Reviewers' Comments:

Reviewer #1 (Remarks to the Author):

Teasing apart pleiotropy between MDD and physical comorbidities.
MDD teased apart using 4 clusters of physical disease: cardiovascular (AF, CAD, HF, Stroke), metabolic (HDL, Metabolic syndrome, T2D, triglycerides), gastrointestinal (GORD, gallstone, IBS, PUD), immune (AD, asthma, IBD, MS).
This is a quality paper of potential great impact and interest, but I do have some questions and concerns.

Three clusters have independent associations with MDD. How much is this driven by power considerations in the immune cluster? It would be helpful to quantify this. Judging by Table 1, they have much smaller sample size and notably lower average Z value (and this is pretty apparent in most figures and data, including locus discovery in the multivariate GWAS).

Some basic but feasible and robust approaches (ldsc, gSEM, PheWAS) with some more innovative/underutilized innovation through multivariate GWAS and biological interpretation through biological annotations.

How was the HDL trait handled in the gSEM model? Was the trait inverted (it looks like no)? If not, how did this influence the interpretation of the metabolic cluster and the overall latent factor? Did you see a difference in how you identified Heywood cases in this cluster in particular? In Figure 2, I didn’t understand why GSD was the only trait co-loaded onto two factors?

Was an exploratory factor analysis run for the same traits? Would be curious to see what the data supports in a hypothesis-free approach, in comparison.

The initial trait selection is a little opaque and hard to understand (but could be resolved with a little more explanation).

Could more be said about the selection criteria for the 29 traits initially selected at the outset for ldsc? Most of the informative traits selected would have survived quite a broad rG scan, while some of those that fail seemed to be obviously short of the "z-score > 7" criteria. For example, chronic pancreatitis and nausea had obviously quite high SE in relation to the reported SNP h2, low zscores couldn't have been surprising. How was this criterion chosen? How were the authors certain they utilized the largest, latest, and most appropriate datasets?

Minor comments:

It's appreciated and elegant to use a consistent color code for clusters for most of the manuscript, but consider describing color coding explicitly in each figure legend (or at least the first one).

Line 41-42, Bonferroni correction less clear than it could be. Just state directly that 19 traits with SNP h2 Z > 7 were corrected for.

Als et al. is a great paper but not the latest/largest given the Cell PGC paper published early this year. Just pointing it out, not expecting much difference in outcome esp. without the 23andMe portion of the sample. It's understandable that data access is a challenge, but why no 23andMe?

Reviewer #2 (Remarks to the Author):

Woodward et al. studies pleiotropy between MDD and 4 different disease types: cardiovascular diseases (CVDs), metabolic diseases, gastrointestinal diseases, and immune-related diseases. The study is based on secondary analyses of GWAS summary statistics. Woodward and colleagues' model pleiotropy using structural equation modelling (SEM) based on the genetic covariance between the traits fitted using GenomicSEM. They regress MDD on latent factors representing the 4 disease types and find that gastrointestinal diseases account for most of the genetic covariance between MDD and physical diseases and conclude that that the gut-brain axis is key in MDD comorbidities. They also fit second order models per disease type to model shared genetic liability between MDD and the disease type and map shared genetic liability to genes, pathways, and tissues. This is an important topic, and the study is well done. I have some comments.

1. The regression model includes 4 latent factors representing different types of disease traits: CVD, metabolic, gastrointestinal, and immune-related. What's the interpretation of model parameters given plausible DAGs? For instance, the manuscript and previous papers link MDD to Type 2 diabetes, triglycerides etc., but these traits are in turn strongly linked to subsequent CVD risk. Are MDD–CVD effects attenuated because this potential mediator (i.e. the metabolic traits) is included? I think that incorporating plausible DAGs in analyses and interpretation would improve the manuscript.

2. The traits underlying the clusters have very different characteristics. For instance, CVD and metabolic traits have lower polygenicity than MDD and gastrointestinal traits (MDD, CVD and metabolic trait polygenicity can be found in Extended Data Figure 2 in PMID: 39215135, polygenicity for IBS in Figure 2 in PMID: 37528461). MDD and GI traits are also more prevalent in the population (especially for younger people). How does that affect the results and interpretation?

3. The traits are different in terms of etiology. CVD has a well understood and described etiology related to lipoproteins, triglycerides, and inflammation, factors which are convincingly linked with MDD in numerous studies (PMID: 39801616). So, a common hypothesis about the MDD–CVD link is that it is mediated by these immunometabolic factors. How does that story relate to the findings of the manuscript? Especially keeping in mind that the etiology of functional GI traits is not well understood.

4. How does power of individual GWASs impact the SEM model and results?

5. MDD and functional GI traits both show strong genetic correlations with behavioral/psychosocial traits (both GORD and MDD show around -0.4 genetic correlation with educational attainment for instance) and both are linked to smoking and alcohol, etc. Is the link between functional GI traits and MDD related to these factors? Childhood adversity might also be an important shared factor.

6. The discussion seems quite general and does not give very specific conclusions/consequences of the study. For instance, based on the results, what specific pathophysiological mechanisms link MDD with functional GI disorders? What specific hypotheses are generated that might be investigated using mouse and cellular models? Are there any viable drug targets that are discovered?

7. The manuscript explains that the large number of independent loci for the MDD-metabolic factor is due to the fact that metabolic diseases are more polygenic than others:
"This cluster's large number of loci highlights the strong polygenicity and statistical power of the univariate metabolic GWAS." Lines 84-86.
However, metabolic traits are usually described to be less polygenic than behavioural traits. In Extended Data Fig 2 in PMID: 39215135 triglycerides, type 2 diabetes, HDL etc. has much lower polygenicity than MDD, educational attainment etc. and according to PMID: 37528461 at least IBS of the gastrointestinal diseases (I'm suspecting that GORD is also highly polygenic) have similar polygenicity to MDD.

Minor comments:

1. It would be nice to see the psychiatric genomics consortium work being used (PMID: 39814019).

2. It would be good to add number of cases to Table 1.

Reviewer #3 (Remarks to the Author):

This study investigated the shared genetic component between four groups of physical diseases (cardiovascular, metabolic, immune, gastrointestinal) and major depressive disorder (MDD).

Analyses were organised in different steps, encompassing genomic structural equation modelling (SEM), multivariate GWAS, phenome-wide association study, biological annotations.

The results suggest that the gastrointestinal cluster has the strongest association with MDD, and that there are independent loci behind the shared genetic component of each disease group and MDD.

The high comorbidity and shared pathogenetic mechanisms between MDD and physical disorders raise interest toward the study of the genetic mechanisms involved. This study provides new and relevant findings in this field, however there are some limitations and possible concerns, as explained below.

Specific comments:

- The factor loadings showed in Figure 3 do not completely correspond to those reported in the supplementary results.

- The negative association between the metabolic factor and MDD should be discussed, as it is unexpected and counterintuitive considering the existing literature. This finding contributes together with the following observation to raise some potential concerns about the robustness of the proposed factor model.

- The model proposed and investigated in several downstream analyses shows the gastrointestinal cluster as the one with the strongest association with MDD, with the immune cluster not showing independent association with MDD. However, when including traits with Z SNP-h2 > 4, the immune cluster explains the largest portion of MDD variance and has the strongest correlation with MDD, while the gastrointestinal group has no longer independent association. The authors discard this model with the justification that is shows lower power, and that “the immune cluster was heavily manipulated”. This choice should be better discussed, better explaining in which ways “the immune cluster was heavily manipulated”, as the model fit looks similar to other latent factors and the strong correlation between ASTH and AD was present also in the model including only traits with Z SNP-h2 > 7. A further reason to address this point is that pathogenetic mechanisms involving the immune system have been reported in MDD (e.g., PMID 31238086, 34724041, 32553197) and that the instability in the effect of the gastrointestinal factor between the two approaches may rise some concerns.

- The authors suggest that the gastrointestinal cluster represents the group of physical diseases with the strongest association with MDD. Looking at several analyses, gastrointestinal diseases also seem the group with the greatest shared genetics with the other diseases of interest, e.g., looking at the associations in figure 3 and the number of non-shared ACAT hits in figure 5A (gastro-MDD has 88/406, while other diseases-MDD pairs all have higher proportion of non-shared hits). The pheWAS analysis also show notable overlap of the identified traits among gastro-MDD and the others disease-MDD pairs, as well as cell-type specific gene associations, suggesting non-specific mechanisms. The interpretation of these findings should be expanded, considering also that the emergence of the gastrointestinal factor looked relatively unexpected, particularly compared to cardiovascular and metabolic factors, and it is not clear what could be driving this result.

- Latent causal variable analysis did not support causality (supplementary results), however this is not clear in the main manuscript text. Given the risk that CAUSE results are inflated, statements about causal effects should be much more cautious in the main manuscript text.

- The GWAS of MDD used in this study included population-based samples (PMID 37464041), e.g., iPSYCH, where different diagnoses can be reported for the same individual over time and there is no structured assessment. The possible implications of this should be discussed, including the impact on the results.

- The inclusion of GWASs of European ancestry only is an important limitation.

Minor comments:

- “[…] and identify tissues with a significant effect across tissues” should likely be features (or genes) with a significant effect across tissues.

- “anxiolytics (drugs used to treat anxiety and/or depression)”: anxiolytics have no indication for the treatment of depression.

Version 1:

Decision Letter:

Our ref: NG-A69310R

29th May 2026

Dear Damian,

Your revised manuscript "Dissecting Pleiotropy Between Major Depressive Disorder and Physical Disease Comorbidities" (NG-A69310R) has been seen by the original referees. As you will see from their comments below, they are satisfied with the revision and have no further requests, and therefore we will be happy in principle to publish your study in Nature Genetics as an Article pending final revisions to comply with our editorial and formatting guidelines.

We are now performing detailed checks on your paper, and we will send you a checklist detailing our editorial and formatting requirements soon. Please do not upload the final materials or make any revisions until you receive this additional information from us.

Thank you again for your interest in Nature Genetics. Please do not hesitate to contact me if you have any questions.

Sincerely,
Kyle

Kyle Vogan, PhD
Senior Editor
Nature Genetics
https://orcid.org/0000-0001-9565-9665

Reviewer #1 (Remarks to the Author):

The authors have addressed my concerns.

Reviewer #2 (Remarks to the Author):

The authors have done an excellent job with the revision. This is an interesting and important paper.

Reviewer #3 (Remarks to the Author):

The authors addressed my previous comments.

Open Access This Peer Review File is licensed under a Creative Commons Attribution 4.0 International License, which permits use, sharing, adaptation, distribution and reproduction in any medium or format, as long as you give appropriate credit to the original author(s) and the source, provide a link to the Creative Commons license, and indicate if changes were made.
In cases where reviewers are anonymous, credit should be given to 'Anonymous Referee' and the source.
The images or other third party material in this Peer Review File are included in the article's Creative Commons license, unless indicated otherwise in a credit line to the material. If material is not included in the article's Creative Commons license and your intended use is not permitted by statutory regulation or exceeds the permitted use, you will need to obtain permission directly from the copyright holder.
To view a copy of this license, visit https://creativecommons.org/licenses/by/4.0/

We thank the reviewer for their careful consideration and constructive feedback. Based on their comments, we have added additional analysis and expanded the discussion. We believe the inclusion of the additional EFA analyses and further information on power and trait selection has strengthened the manuscript.

Referee #1:

Three clusters have independent associations with MDD. How much is this driven by power considerations in the immune cluster? It would be helpful to quantify this. Judging by Table 1, they have much smaller sample size and notably lower average Z value (and this is pretty apparent in most figures and data, including locus discovery in the multivariate GWAS).

This is a good point, and we added more discussion and provided several metrics to demonstrate the power differences (Supplementary Table 19). Overall, the immune cluster has comparatively lower trait power and smaller shared variance, which reflects locus discovery. Additionally, we discuss several factors and considerations influencing the interpretation of the immune cluster and its results.

Supplementary Results: Lines 313 to 333

5.0 Power Quantification

We next explored differences in power across the disease clusters (Supplementary Table 19). First, we examined the power of the individual traits within the cluster using their h^2_{SNP} Z scores. Second, we investigated the genetic overlap among traits within each disease cluster, as reflected in the factor structure, i.e., the greater the shared genetic variance, the greater the variance captured by the latent factor. Together, the individual trait power and the extent of shared variance will influence the overall power of each physical disease cluster.

On average, the metabolic cluster had the highest h^2_{SNP} Z (mean = 16.83) and the strongest factor loadings (mean $|\lambda|$ = 0.80), suggesting that this latent factor captures a relatively large proportion of shared variance across traits. Consistent with this stronger shared signal and trait power, the metabolic-MDD second-order factor yielded the largest number of loci (N = 537). Both CVD and gastrointestinal disease clusters showed broadly

similar signal strength, as indicated by the input traits (CVD: mean h^2_{SNP} Z = 13.01; Gastro: mean h^2_{SNP} Z = 14.11) and genomic SEM parameters (CVD: mean | λ | = 0.69; Gastro: mean | λ | = 0.67). Accordingly, they yielded a similar number of loci in the multivariate GWAS (CVD: N = 172; Gastro: N = 170). Lastly, the immune cluster has the lowest average h^2_{SNP} Z (mean = 10.95) and the weakest factor loadings (mean | λ | = 0.35), indicating that the latent factor captured the least shared variance across traits. Nevertheless, the immune multivariate GWAS identified 140 loci, indicating that although the signal is comparatively weaker than the other clusters, it was not absent. Overall, these findings show differences in signal strength and shared variance across clusters, which reflect differences in locus discovery.

Manuscript: Lines 243 to 252

It is important to consider the differences in the power of disease cluster traits, which are influenced by effective sample size and SNP heritability. In genomic SEM, power impacts the precision of LDSC inputs, factor measurements, and statistical significance of path coefficients. We observed model instability at a lower trait Z-score cutoff threshold (h^2_{SNP} Z > 4; Supplementary Results, Section 3.0), and, notably, the immune cluster became insignificant in the joint model, a change that may partly reflect its lower power. Furthermore, differences in the number of shared loci or enriched functional annotations should not be interpreted as evidence for stronger biological overlap with MDD, as these patterns may reflect variations in GWAS power. For example, the metabolic-MDD cluster showed strong enrichment in gene-set analysis and identified the most loci; however, comparatively, the metabolic cluster has the most power.

Manuscript: Lines 257 to 272

Lastly, the immune cluster was not independently associated with MDD in the joint model. This does not implicate immune mechanisms as unrelated to MDD; rather, this cluster's association with MDD is shared with other factors, particularly the gastrointestinal factor, which could reflect the strong link between gastrointestinal disorders and immune mechanisms[1]. However, we note several limitations of the immune cluster that may influence these findings. This cluster had the smallest shared variance among the individual traits, as evidenced by its weaker genetic correlations and factor loadings, consistent with a less uniform shared signal. A potential cause is the

heterogeneous nature of immune disorders, with various subtypes, immune responses, and effects on different tissues and organs. Cross-disorder genetic analyses have revealed distinct clusters of genetic associations in immune-mediated diseases[2] as well as psychiatric factors associated with immune subtypes to different degrees[3]. Additionally, the MHC region was removed from genomic SEM and LDSC due to the complex LD structure, which may also influence the results of the immune cluster, given that HLA type is often the strongest common genetic risk factor for immune-driven disorders. With larger sample sizes for immune traits, future work should examine the various immune subtypes and the influence of the MHC region on physical disease-MDD comorbidities.

How was the HDL trait handled in the gSEM model? Was the trait inverted (it looks like no)? If not, how did this influence the interpretation of the metabolic cluster and the overall latent factor? Did you see a difference in how you identified Heywood cases in this cluster in particular? In Figure 2, I didn't understand why GSD was the only trait co-loaded onto two factors?

High-density lipoprotein (HDL) is negatively correlated with MDD and other traits (Figure 2). This is biologically concordant, as HDL is generally protective, with higher HDL levels associated with a lower risk of metabolic and cardiovascular diseases. This is reflected in the genomic SEM modelling, with the negative factor loading indicating that higher metabolic factor liability is associated with lower HDL. Inverting the trait to switch the sign of the factor loading to positive would produce the same interpretation, associated loci, model fit, and model parameters. An additional comment on this has been added to improve clarity.

Supplementary Results Lines 58 to 61:

HDL had the only negative factor loading across clusters, with -0.73. The negative loading indicates that higher metabolic factor liability is associated with lower HDL levels. This loading is biologically concordant, as lower HDL levels are associated with an increased risk of MSYN, T2D, and CVD[4,5].

The cross-loading factor of GSD was required for two reasons. First, without cross-loading, the residual covariance between each metabolic trait and GSD in the four-factor model was large (residual correlations > 0.10). This indicates that the model did not accurately capture the relationship between GSD and metabolic traits. Secondly, it is biologically plausible that GSD loads on both factors, i.e., GSD is considered a gastrointestinal phenotype with a strong association with metabolic risk (T2D and MSYN). This was verified using the cross-loading approach. By loading GSD onto the metabolic factor, residual correlations were reduced, and model fit significantly improved, suggesting that the genetic variance of GSD was better explained by a combination of metabolic and gastrointestinal liability. No other trait showed high residual variance across the other disease cluster traits. This has been elaborated on in the methods and results.

Methods: Lines 326 to 328.

Traits that showed high residual correlations across other disease clusters (>0.10) were allowed to load onto multiple factors if this improved model fit, as assessed by a chi-square test and CFI/SRMR estimates.

Supplementary Results: Lines 90 to 97.

However, GSD showed a high level of residual covariance among metabolic traits (residual correlation > 0.10). GSD is plausibly a gastrointestinal phenotype with a strong association with metabolic risk, including diabetes[6] and MSYN[7]. Therefore, GSD was loaded onto both metabolic ($\lambda = 0.37$) and gastrointestinal disease ($\lambda = 0.31$) factors. This significantly improved model fit ($\Delta\chi^2$ (1) = 635.46, $P = 3.26 \times 10^{-140}$), with a small improvement in CFI (ΔCFI = 0.015), and lower SRMR (ΔSRMR = -0.009), suggesting that the genetic variance of GSD was better explained by a combination of metabolic and gastrointestinal liability (CFI = 0.918; SRMR = 0.055). No other trait demonstrated high residual variance across a range of cluster traits.

Was an exploratory factor analysis run for the same traits? Would be curious to see what the data supports in a hypothesis-free approach, in comparison.

Thank you for this suggestion; we have added this analysis to compare the fit of an EFA with the fit of our hypothesised model. We demonstrated that the EFA model was similar to the four-factor CFA model identified.

Methods: Lines 329 to 334.

Exploratory Factor Analysis. We performed an exploratory factor analysis (EFA) to compare the number of factors and the loading patterns suggested by the data, without imposing the latent structure specified in the confirmatory model. We used the LDSC covariance matrix and the *fa* function in the *psych* R package[8], with oblique rotation (oblimin) and maximum likelihood (ml) settings. Factors with eigenvalues greater than one were extracted, and a loading cutoff of 0.30 was used to define strong loadings.

Supplementary Results: Lines 110 to 128.

2.6 Exploratory Factor Analysis

We conducted an exploratory factor analysis (EFA) to assess the latent structure and factor loadings. EFA indicated a five-factor model based on eigenvalues greater than one (Supplementary Figure 2a). The five-factor EFA model had similar metabolic, gastrointestinal, and immune groupings to the identified confirmatory model (Supplementary Table 4). The addition of the fifth factor split CVD traits into two clusters, creating subdivisions of the CVD cluster with only two traits loading onto each. The two CVD factors had the highest correlation across factors at 0.51 (Supplementary Figure 2b).

We next examined a four-factor exploratory solution to compare to the five-factor EFA (Supplementary Figure 2c; Supplementary Table 4). The four-factor EFA exhibited disease traits clustering closely with the confirmatory model. The traits showed clear groupings within the suggested factors, albeit with loadings below the cutoff in the immune cluster. When comparing the two EFA models, we observed minor improvements in fit indices with five factors (Supplementary Table 5; Five-factor: χ^2 = 2.298; fit.off = 0.985, df-RSMR = 0.056; Four-factor: χ^2 = 3.931, fit.off = 0.980, df-RSMR = 0.058). However, the four-factor model (BIC = -167.97) demonstrated superior fit to the five-factor model (BIC = -136.33),

suggesting that the five-factor model did not provide sufficient improvement to justify the added model complexity. The fifth factor may represent a subdivision of CVD genetic liability rather than a broader physical disease domain.

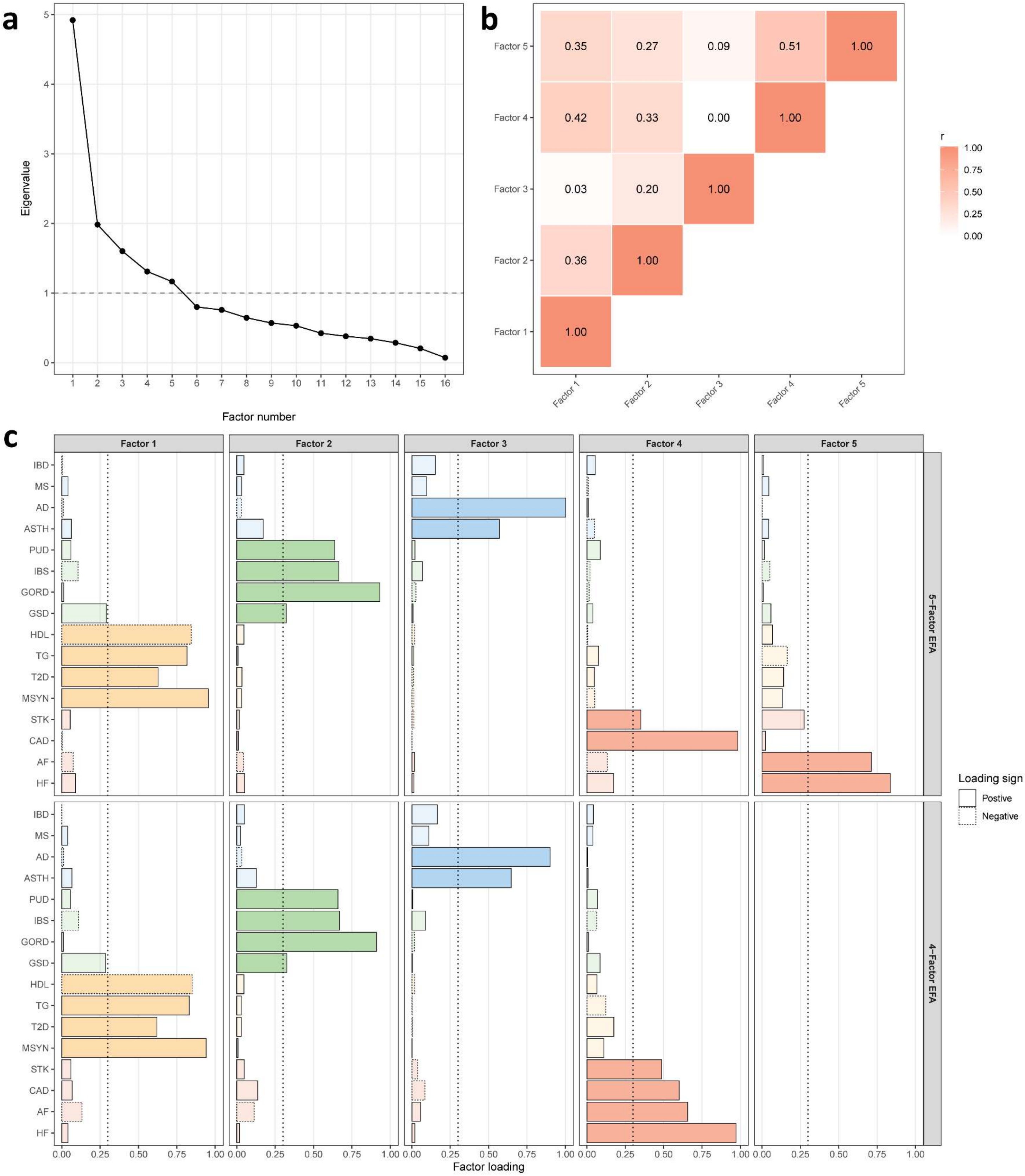

Supplementary Figure 2. Exploratory Factor Analysis (EFA) of the trait covariance structure. **a**. Scree plot of eigenvalues and the corresponding number of factors. **b.** The factor correlations of the five-factor exploratory solution. **c.** Comparing rotated factor loadings of a five-factor and four-factor EFA. Absolute factor loadings of each trait on the models are illustrated. Stronger loadings above the prespecified cutoff of 0.30 are emphasised, with negative loadings outlined in dotted lines. Acronyms: Atrial Fibrillation (AF), Coronary Artery Disease (CAD), Heart Failure (HF), Stroke (STK), Metabolic Syndrome (MSYN), Type 2 Diabetes (T2D), Triglycerides (TG), High-density lipoprotein cholesterol (HDL), Gallstone Disease (GSD), Gastro-oesophageal Reflux Disease (GORD), Irritable Bowel Syndrome (IBS), Peptic Ulcer Disease (PUD), Asthma (ASTH), Atopic Dermatitis (AD), Multiple Sclerosis (MS), Inflammatory Bowel Disease (IBD).

The initial trait selection is a little opaque and hard to understand (but could be resolved with a little more explanation). Could more be said about the selection criteria for the 29 traits initially selected at the outset for ldsc? Most of the informative traits selected would have survived quite a broad rG scan, while some of those that fail seemed to be obviously short of the "z-score > 7" criteria. For example, chronic pancreatitis and nausea had obviously quite high SE in relation to the reported SNP h2, low zscores couldn't have been surprising. How was this criterion chosen? How were the authors certain they utilized the largest, latest, and most appropriate datasets?

The SEs of the SNP-h2 and Z-scores indeed represent similar information, with SE reflecting trait sample size, whereas Z reflects the sample size and heritability. We followed the Z-score cut-off recommended by LDSC[9] to obtain reliable estimates, consistent with common practice in genomic SEM literature[3,10,11] . We tested this minimum threshold and a higher threshold in LDSC and genomic SEM (Supplementary Results), and we have provided more information on trait selection.

Supplementary Results: Lines 1 to 37

1.0 Data Selection

We compiled a broad set of candidate traits across the four clusters, drawing on previous research linking traits to disease clusters and MDD[12]. For each trait, we downloaded publicly available GWAS in European ancestry from data repositories and consortia (see Supplementary Table 1 for details). To select robust, well-powered GWAS, preference was given to studies with the largest number of cases/total sample sizes, the most stringent phenotypic definition, and the greatest number of identified associations. The initial trait candidate approach was intentionally inclusive to explore potential associations with physical diseases and MDD. These were then filtered for downstream modelling using predefined statistical criteria. We used LDSC to filter out traits that were not significantly correlated with MDD or had low power using the recommended minimum threshold to obtain reliable estimates of genetic overlap (h^2_{SNP} $Z > 4$)[9].

1.1 Cardiovascular Cluster

Trait selection for CVD represented related yet distinct CVD manifestations to capture broad vascular and cardiac genetic liability. These reflect cardiac rhythm disturbances (AF), atherosclerotic vascular phenotypes (CAD, PAD), cerebrovascular (STK), and heart failure (HF). CVD traits closely followed Bergstedt et al. (2024), who developed a CVD-MDD model and identified shared biological mechanisms underlying CVD-MDD comorbidity[13].

1.2 Metabolic Cluster

This cluster aimed to capture metabolic dysfunction, biologically spanning lipid and glucose dysregulation, often prominent in MDD patients[14,15]. To explore these metabolic/systemic physiological states (e.g. lipid fractions, glycaemic endpoints), we included multiple metabolic biomarkers (HDL, LDL, TG), a syndrome-level construct (MSYN), and a metabolic disease endpoint (T2D).

1.3 Gastrointestinal Cluster

This physical disease cluster examined gastrointestinal disease burden across both functional and non-functional gut phenotypes. Traits for this cluster were selected to span multiple gastrointestinal regions and mechanisms. These included functionally related gastrointestinal phenotypes (IBS, GORD, NE), small-intestinal enteropathy disease (CD), disorders affecting the upper gastrointestinal tract (GORD, PUD), and hepatobiliary mechanisms (GSD).

1.4 Immune Cluster

The aim of this cluster was to identify potential immune-mediated disease liability associated with MDD. We included a wide range of immune traits to reflect the diversity of immune responses, including allergic/atopic inflammation (ASTH, AD), systemic autoimmune (RA, SLE), chronic inflammatory conditions (IBD, CP, PSV), organ-specific autoimmunity (T1D, HD), hepatobiliary autoimmune disorders (PBC, PSC) and CNS autoimmunity (MS). This would avoid narrow representation and capture a range of immune mechanisms and systems that may be shared with MDD, as well as whether genetic overlap is concentrated in immune subtypes.

It’s appreciated and elegant to use a consistent color code for clusters for most of the manuscript, but consider describing color coding explicitly in each figure legend (or at least the first one).

A colour coding description has been added to the figures.

Line 41-42, Bonferroni correction less clear than it could be. Just state directly that 19 traits with SNP h2 Z > 7 were corrected for.

We have made the suggested changed to the manuscript.

Manuscript: Lines 51 to 54

We selected well-powered physical traits (h^2_{SNP} Z-score > 7) for each disease cluster using Linkage Disequilibrium Score Regression (LDSC). Of these traits, 16 showed a significant genetic correlation with MDD after correcting for multiple testing for 19 traits ($\alpha < 2.63 \times 10^{-3}$; 0.05/19) and were retained for downstream analysis (Figure 2; Table 1).

Als et al. is a great paper but not the latest/largest given the Cell PGC paper published early this year. Just pointing it out, not expecting much difference in outcome esp. without the 23andMe portion of the sample. It's understandable that data access is a challenge, but why no 23andMe?

We were not able to access the 23andMe summary statistics as our application was delayed due to the 23andMe administrative problems. We agree that the outcomes are expected to be only minimally different.

We are grateful to the reviewer for their thoughtful feedback and helpful suggestions. The suggestion to include DAG's, with the social/behavioural trait models, enabled a more detailed examination of the disease clusters. Additionally, the expanded biological interpretation in the discussion has helped to contextualise the results.

Referee #2:

1. **The regression model includes 4 latent factors representing different types of disease traits: CVD, metabolic, gastrointestinal, and immune-related. What's the interpretation of model parameters given plausible DAGs? For instance, the manuscript and previous papers link MDD to Type 2 diabetes, triglycerides etc., but these traits are in turn strongly linked to subsequent CVD risk. Are MDD–CVD effects attenuated because this potential mediator (i.e. the metabolic traits) is included? I think that incorporating plausible DAGs in analyses and interpretation would improve the manuscript.**

Thank you for this suggestion. To further interpret the relationship between disease clusters and MDD, we estimated plausible DAGs informed by previous analyses.

We fit mediation models in genomic SEM, focusing on the gastrointestinal cluster. We examined whether it served as a potential mediator of the other clusters, as this group demonstrated the strongest association in the joint model. The analysis demonstrated the gastrointestinal cluster as a potential mediator of the relationship between CVD, metabolic, and immune factors and MDD, although our analyses do not provide information on the causal effect.

In addition, we used mediation models to examine the driver of the negative association between the metabolic cluster and MDD in the joint model. The metabolic group shows a positive association, but this becomes negative in the four-factor model.

Methods: Lines 329 to 334

Directed Acyclic Graphs. To examine potential mediators between physical disease clusters and MDD, we constructed mediation models in genomic SEM. These models were based on hypothesised relationships identified in previous analyses. We used

thresholds of CFI > 0.90 and SRMR < 0.10 for acceptable fit with models estimated using diagonally weighted least squares.

Supplementary Results: Lines 129 to 153

2.7. Directed Acyclic Graphs

We constructed directed acyclic graphs (DAGs) in genomic SEM to further explore the relationships between physical disease clusters. We note that these models in genomic SEM are best interpreted as variance decompositions that distinguish shared from cluster-specific components, rather than identifying a causal direction. In addition, attenuation or an insignificant association does not mean an absence of shared biology; instead, it is interpreted as a redundancy or redistribution of shared variance.

As we observed the gastrointestinal cluster as the predominant factor in the joint model, we examined whether this alone was explaining the attenuation of the other factors (Supplementary Table 6; Supplementary Figure 3a). We observed that, for all other factors, the direct path to MDD became insignificant when the gastrointestinal factor was included as a potential mediator. These models suggest the variance explained by CVD, metabolic, and immune factors in MDD is shared with the gastrointestinal cluster.

Next, we investigated DAGs for the metabolic factor to examine the driver of the cluster's negative association with MDD. We included each factor as a potential mediator (Supplementary Table 7; Supplementary Figure 3b) and performed a leave-one-out analysis as a sensitivity check to assess whether a single trait was inverting this association (Supplementary Table 8). We note that the metabolic-CVD mediation model was marginally below the conventional CFI threshold (CFI = 0.894), but had an acceptable SRMR (0.066), and was therefore retained for DAG purposes. Our findings indicated that the inverse association is driven by the gastrointestinal factor and is not attributable to a single trait in either cluster (gastrointestinal or metabolic). Additionally, this association is no longer significant when accounting for gastrointestinal alone. This should not be interpreted as metabolic disease being protective against MDD, but rather that MDD-relevant metabolic liability is largely shared within the gastrointestinal cluster, with a weak inverse conditional association when all four clusters are included.

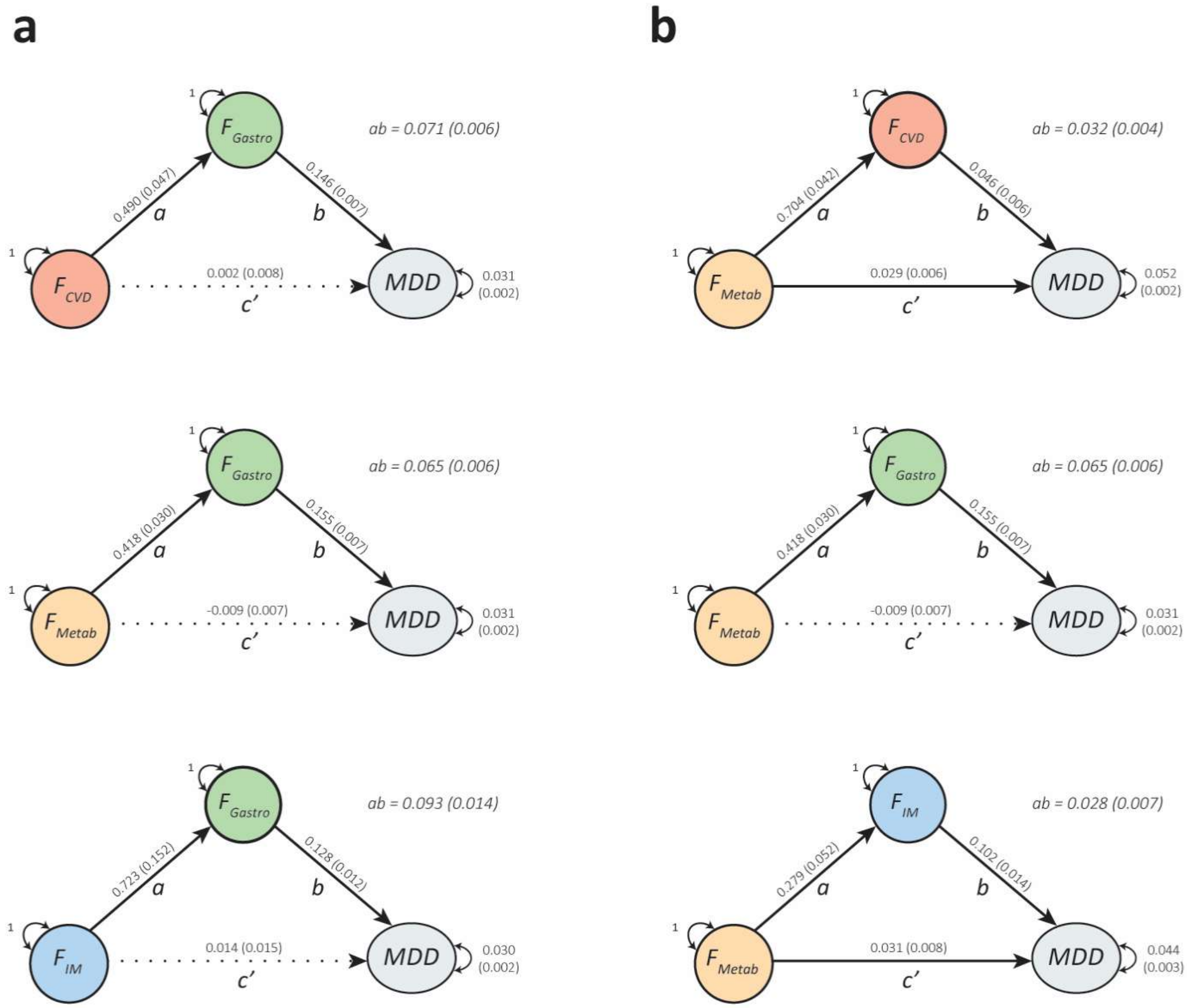

Supplementary Figure 3. Directed Acyclic Graphs (DAGs) examining gastrointestinal and metabolic clusters. a. DAGs investigating the impact of gastrointestinal cluster on the association between other disease clusters and MDD. **b.** DAGS exploring the driver of the negative association of the metabolic cluster and MDD. Dotted lines indicate an insignificant path (P > 0.05). The letters denote separate paths, the direct path (c') and the indirect path (a*b). Unstandardised estimates are described. Acronyms: Cardiovascular (CVD), metabolic (Metab), gastrointestinal (gastro), immune (IM), major depressive disorder (MDD).

2. **The traits underlying the clusters have very different characteristics. For instance, CVD and metabolic traits have lower polygenicity than MDD and gastrointestinal traits (MDD, CVD and metabolic trait polygenicity can be found in Extended Data Figure 2 in PMID: 39215135, polygenicity for IBS in Figure 2 in PMID: 37528461). MDD and GI traits are also more prevalent in the population (especially for younger people). How does that affect the results and interpretation?**

A basic assumption of LDSC (and thus, Genomic SEM) is that traits are polygenic and follow a uniform distribution of effect sizes. While LDSC is derived under a polygenic model, simulations from the original LDSC paper[9] show that the slope and intercept remain unbiased even when only a small fraction of variants are causal (LDSC paper: Supplementary Figures 3-4)[9]. Rather, the main consequence is increased sampling variance of the estimates (i.e. large standard errors; Supplementary Figure 5)[9]. There may be situations in which bias would be expected (e.g., traits with extreme genetic architectures, such as when all causal variants are rare). As you note, there are indeed differences in polygenicity among traits in our study, but none of the traits we include follow an extreme/rare-variant architecture. Hence, it is expected that differences in polygenicity will only affect the standard errors of the estimates but will remain unbiased. Further, Genomic SEM explicitly accounts for differences in precision across elements of the genetic covariance matrix by using its associated sampling covariance matrix (V) to estimate accurate standard errors. Thus, differing levels of polygenicity across traits would only be expected to result in minor differences in precision, but not the accuracy of model parameters or subsequent analyses.

We added as a limitation that our estimates are restricted to common genetic variants and are based on the assumption of a uniform distribution of effect sizes.

Manuscript: Lines 241 to 242

Fourth, the results are restricted to common genetic variants and assume a uniform distribution of effect sizes.

Disease prevalence was accounted for in genomic SEM and genetic correlations, as it is used as input to LDSC to estimate the general population risk for binary traits and to

enable comparisons of heritability estimates. GWAS estimates represent the age distribution and ascertainment of the contributing cohorts. Consequently, the interpretation of downstream results reflects a mixture of populations across the underlying GWASs for each second-order factor. Differences in prevalence across age groups is an interesting point, however, cannot be investigated with the current datasets.

3. The traits are different in terms of etiology. CVD has a well understood and described etiology related to lipoproteins, triglycerides, and inflammation, factors which are convincingly linked with MDD in numerous studies (PMID: 39801616). So, a common hypothesis about the MDD–CVD link is that it is mediated by these immunometabolic factors. How does that story relate to the findings of the manuscript? Especially keeping in mind that the etiology of functional GI traits is not well understood.

We have elaborated on this important question in the discussion. The results suggest immune-metabolic mediation is not separable from gastrointestinal liability in our multivariate genetic model. Rather than a single linear direction, we observe a multisystem, shared architecture across clusters that share substantial variance. In this framework, our model demonstrated that the MDD-relevant component of metabolic and immune liability is coupled to and statistically captured by gastrointestinal liability. Although functional gastrointestinal disorders are less well understood, they may serve as proximal markers of gut-brain processes through which broader liabilities may operate.

Manuscript: Lines 190 to 199

In line with prior research[16], we demonstrate a shared genetic basis underlying the gut-brain axis, MDD and gastrointestinal diseases. Our results expand on these findings, showing that this is likely due to psychopathological pathways related to functional gastrointestinal diseases that are associated with other disease systems. For example, a mechanistic hypothesis for CVD-MDD comorbidity is that it is mediated by immune-metabolic pathways[17]. Our shared-liability framework suggests immune-metabolic genetic liability largely overlaps with the gastrointestinal cluster rather than contributing independent components. In other words, gut-brain mechanisms may act and partially overlap with immune-metabolic pathways. The gut-brain axis has exhibited connections with CVD[18], diabetes[19], metabolic syndrome[20] and immune diseases[21]. We observe that this axis may influence the genetic relationship of these diseases with MDD.

4. How does power of individual GWASs impact the SEM model and results?

This is an excellent point, and we have added more discussion and results regarding the influence of power. Overall, the metabolic cluster has the highest power/shared variance, and the immune cluster has the lowest power/smaller shared variance, which is reflected in locus discovery. Importantly, we have implemented the recommended cutoffs for LDSC/genomic SEM and selected well-powered traits for each disease cluster.

Supplementary Results: Lines 313 to 333

5.0 Power Quantification

We next explored differences in power across the disease clusters (Supplementary Table 19). First, we examined the power of the individual traits within the cluster using their h^2_{SNP} Z scores. Second, we investigated the genetic overlap among traits within each disease cluster, as reflected in the factor structure, i.e., the greater the shared genetic variance, the greater the variance captured by the latent factor. Together, the individual trait power and the extent of shared variance will influence the overall power of each physical disease cluster.

On average, the metabolic cluster had the highest h^2_{SNP} Z (mean = 16.83) and the strongest factor loadings (mean | λ | = 0.80), suggesting that this latent factor captures a relatively large proportion of shared variance across traits. Consistent with this stronger shared signal and trait power, the metabolic-MDD second-order factor yielded the largest number of loci (N = 537). Both CVD and gastrointestinal disease clusters showed broadly similar signal strength, as indicated by the input traits (CVD: mean h^2_{SNP} Z = 13.01; Gastro: mean h^2_{SNP} Z = 14.11) and genomic SEM parameters (CVD: mean | λ | = 0.69; Gastro: mean | λ | = 0.67). Accordingly, they yielded a similar number of loci in the multivariate GWAS (CVD: N = 172; Gastro: N = 170). Lastly, the immune cluster has the lowest average h^2_{SNP} Z (mean = 10.95) and the weakest factor loadings (mean | λ | = 0.35), indicating that the latent factor captured the least shared variance across traits. Nevertheless, the immune multivariate GWAS identified 140 loci, indicating that although the signal is comparatively weaker than the other clusters, it was not absent. Overall, these findings show differences in signal strength and shared variance across clusters, which reflect differences in locus discovery.

Manuscript: Lines 243 to 256

It is important to consider the differences in the power of disease cluster traits, which are influenced by effective sample size and SNP heritability. In genomic SEM, power impacts the precision of LDSC inputs, factor measurements, and statistical significance of path coefficients. We observed model instability at a lower trait Z-score cutoff threshold (h^2_{SNP} Z > 4; Supplementary Results), and, notably, the immune cluster became insignificant in the joint model, a change that may partly reflect its lower power. Furthermore, differences in the number of shared loci or enriched functional annotations should not be interpreted as evidence for stronger biological overlap with MDD, as these patterns may reflect variations in GWAS power. For example, the metabolic-MDD cluster showed strong enrichment in gene-set analysis and identified the most loci; however, comparatively, the metabolic cluster has the most power. Similarly, differences in power with MDD led to more pronounced effects on the second-order factors, despite equivalent loadings. For instance, the metabolic-MDD factor showed a stronger metabolic association, leading to the nervous system and psychiatric enrichment appearing relatively diluted.

5. MDD and functional GI traits both show strong genetic correlations with behavioral/psychosocial traits (both GORD and MDD show around -0.4 genetic correlation with educational attainment for instance) and both are linked to smoking and alcohol, etc. Is the link between functional GI traits and MDD related to these factors? Childhood adversity might also be an important shared factor.

Thank you for raising this; it has prompted an additional analysis examining MDD, physical disease clusters, and social and behavioural traits. We identified that the gastrointestinal cluster was not independent of MDD's associations with several traits. The findings highlight the functional component of the gastrointestinal cluster, capturing shared pathways and risk factors for MDD. These correspond to social factors and behavioural habits, such as educational attainment, high income, sleep behaviours, alcohol use, and physical activity.

Methods: Lines 389 to 397:

Social and Behavioural Traits. Multiple regression was conducted using MDD and the physical disease latent factors to examine associations with social and behavioural traits. Common social and behavioural traits associated with MDD were selected (Supplementary Table 24). We performed LDSC in the genomic SEM R package on these traits, physical disease common factors, and MDD using default parameters. Next, in genomic SEM, we compared two models for each social and behavioural trait. First, MDD is regressed separately on the trait and each factor to estimate a baseline regression coefficient (MDD ~ Trait; MDD ~ Factor). Second, multiple regression models were fitted in which MDD was regressed on both the social/behavioural trait and the physical disease factor (MDD ~ Trait + Factor).

Supplementary Results: Lines 380 to 396

7.0 Social and Behavioural Traits

7.1 Multiple Regression

We examined how the association between physical disease clusters and MDD changes when controlling for social and behavioural traits (Supplementary Figure 10; Supplementary Table 24). This would help establish if the disease cluster associations

with MDD are independent of social and behavioural traits. MDD demonstrated a significant association with each of the social and behavioural traits, with childhood maltreatment (β = 0.55, SE = 0.03, $P = 1.11 \times 10^{-64}$) and high income being the strongest (β = -0.36, SE = 0.03, $P = 1.11 \times 10^{-45}$). The multiple regression models showed a minimal reduction in the physical disease-MDD association. However, we observed attenuations in the paths between the trait and MDD. In particular, the gastrointestinal cluster demonstrated the greatest effect. Physical activity (β = -0.02, SE = 0.04, *P* = 0.53), sleep duration (β = 0.01, SE = 0.02, *P* = 0.46), alcohol use (β = 0.03, SE = 0.03, *P* = 0.25), and high income (β = -0.06, SE = 0.04, *P* = 0.09), became nonsignificant, and MDD associations with educational attainment ($\beta_1 = -0.27 \rightarrow \beta_2 = 0.10$), and childhood maltreatment ($\beta_1 = 0.56 \rightarrow \beta_2 = 0.32$) reduced. The other physical disease groups had minimal reductions. These findings suggest that the gastrointestinal cluster captures shared components linking social/behavioural patterns to MDD liability.

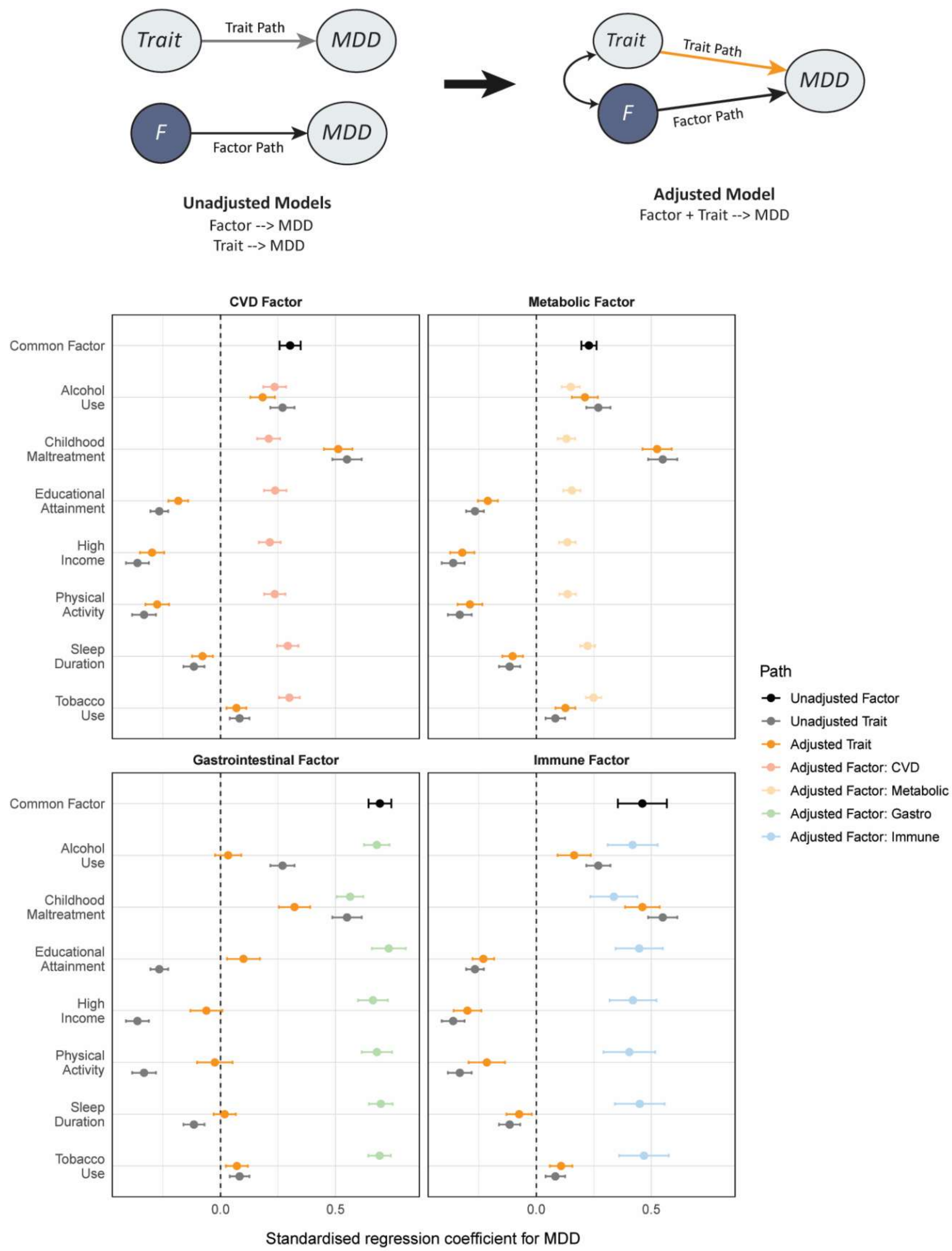

Supplementary Figure 10. Multiple regression analysis of physical disease clusters and social and behavioural factors on major depressive disorder (MDD). Unadjusted models correspond to the association of the disease cluster and trait on MDD, independently (regression coefficient). The adjusted model includes both the social/behavioural trait and the physical disease common factor to examine the impact each trait has on the association. Error bars represent 95% confidence intervals. Acronyms: Cardiovascular (CVD), metabolic (Metab), gastrointestinal (gastro), immune (IM), major depressive disorder (MDD).

6. The discussion seems quite general and does not give very specific conclusions/consequences of the study. For instance, based on the results, what specific pathophysiological mechanisms link MDD with functional GI disorders? What specific hypotheses are generated that might be investigated using mouse and cellular models? Are there any viable drug targets that are discovered?

We have added further discussion of the potential mechanisms, highlighting specific results identified. We note enrichment of dopaminergic pathways in GO gene sets, as well as targets for ATC codes, particularly the D2 receptor. Research suggests that these pathways play essential roles in gut-brain communication and microbiota dysbiosis.

Manuscript: Lines 200 to 217

The enteric nervous system (ENS) is a key component of the gut-brain relationship. The ENS and CNS have shared neurotransmission, signalling cascades, and structural features, which often result in CNS pathology emerging in enteric systems[22]. We observed that gastrointestinal-MDD shared genetic liability was associated with functional gastrointestinal disease, gene regulatory signals in digestive tissues, and drug classes that enhance gut motility (e.g., propulsives). Together with identified neuronal gene sets, these findings suggest that neural mechanisms of gut function may contribute to this genetic overlap and point to ENS-related manifestations. We observed the dopaminergic system as an enriched neurotransmitter system, with particular emphasis on the D2 receptor in both MDD and gastrointestinal liability. This receptor has been shown to be a key communicator between the gut, brain and microbiota[23], is associated with multiple gastrointestinal diseases[24], and shows digestive benefits when targeted with medication[24]. For example, many gastrointestinal treatments (e.g., Domperidone[24]) are D2 receptor inhibitors or antagonists for which we show upregulated expression in gastrointestinal tissues. Additionally, the ENS is tightly coupled with immune-related mechanisms[25] which may contribute to the observed attenuation of the MDD-immune associations. Further research should examine the CNS-ENS relationship in MDD, including immune responses, and the manifestations of MDD-physical disease liability in neurotransmission, particularly dopaminergic pathways.

7. The manuscript explains that the large number of independent loci for the MDD-metabolic factor is due to the fact that metabolic diseases are more polygenic than others: "This cluster's large number of loci highlights the strong polygenicity and statistical power of the univariate metabolic GWAS." Lines 84-86. However, metabolic traits are usually described to be less polygenic than behavioural traits. In Extended Data Fig 2 in PMID: 39215135 triglycerides, type 2 diabetes, HDL etc. has much lower polygenicity than MDD, educational attainment etc. and according to PMID: 37528461 at least IBS of the gastrointestinal diseases (I'm suspecting that GORD is also highly polygenic) have similar polygenicity to MDD.

We agree that metabolic traits are less polygenic than other traits used. We have removed the term "strong polygenicity" from the explanation. The power of the individual traits and the resulting shared variance of the latent factor are likely the drivers of the high number of loci in the metabolic-MDD cluster compared to the other factors (as discussed above).

Minor comments:

1. **It would be nice to see the psychiatric genomics consortium work being used (PMID: 39814019).**

These data were not available at the time we conducted this study. We considered updating all our analyses. However, the increase in cases is only 5%, so we do not believe the inclusion of PGC will significantly affect the results. We were unable to access the 23andMe summary statistics because our application was delayed due to 23andMe administrative issues.

Adams et al. (2025) (excluding 23andMe): N Cases: 412,305; N Controls: 1,588,397.

Als et al. (2023) (excluding 23andMe): N Cases: 394,322; N Controls: 741,438

2. **It would be good to add number of cases to Table 1.**

Cases and controls have been added to Table 1.

We thank the reviewer for their careful review and helpful suggestions. In addressing these comments, we were able to provide clearer framing of the identified models and expand the discussion of the study's limitations and interpretability. We believe these revisions have strengthened the manuscript.

Reviewer #3

- The factor loadings showed in Figure 3 do not completely correspond to those reported in the supplementary results.

We apologise for any confusion. We have checked the factor loadings and those reported in the Supplementary Results and Supplementary Table 3. From our review (conducted by three of the co-authors), we found no discrepancies. Our figures and reporting for these models are based on the "STD_All" column in the genomic SEM output. We have made a correction to make it clear that the factor loadings in Figure 3 correspond to this column in Supplementary Table 3. We have added this to the figure legend. STD_All represents fully standardised parameters from the lavaan output, including any endogenous variables. Compared to the STD_Genotype, which standardises using the genetic variance of the observed traits and is not fully standardised if the latent factor is not itself specified to have a variance of 1.

- The negative association between the metabolic factor and MDD should be discussed, as it is unexpected and counterintuitive considering the existing literature. This finding contributes together with the following observation to raise some potential concerns about the robustness of the proposed factor model.

We observed a positive association between the metabolic factor and MDD in the single-factor model; however, this association turns negative in the joint four-factor model. This indicates that the independent component of the metabolic factor is associated with a small negative effect on MDD and should not be interpreted as metabolic disease being protective against MDD.

We added directed acyclic graph analysis to further examine potential mediators between MDD and physical disease clusters, including investigating the driver of the negative metabolic association.

Methods: Lines 335 to 338

Directed Acyclic Graphs. To examine potential mediators between physical disease clusters and MDD, we constructed mediation models in genomic SEM. These models were based on hypothesised relationships identified in previous analyses. We used thresholds of $CFI > 0.90$ and $SRMR < 0.10$ for acceptable fit with models estimated using diagonally weighted least squares.

Supplementary Results: Lines 129 to 153

2.7. Directed Acyclic Graphs

We constructed directed acyclic graphs (DAGs) in genomic SEM to further explore the relationships between physical disease clusters. We note that these models in genomic SEM are best interpreted as variance decompositions that distinguish shared from cluster-specific components, rather than identifying a causal direction. In addition, attenuation or an insignificant association does not mean an absence of shared biology; instead, it is interpreted as a redundancy or redistribution of shared variance.

As we observed the gastrointestinal cluster as the predominant factor in the joint model, we examined whether this alone was explaining the attenuation of the other factors (Supplementary Table 6; Supplementary Figure 3a). We observed that, for all other

factors, the direct path to MDD became insignificant when the gastrointestinal factor was included as a potential mediator. These models suggest the variance explained by CVD, metabolic, and immune factors in MDD is shared with the gastrointestinal cluster.

Next, we investigated DAGs for the metabolic factor to examine the driver of the cluster's negative association with MDD. We included each factor as a potential mediator (Supplementary Table 7; Supplementary Figure 3b) and performed a leave-one-out analysis as a sensitivity check to assess whether a single trait was inverting this association (Supplementary Table 8). We note that the metabolic-CVD mediation model was marginally below the conventional CFI threshold (CFI = 0.894), but had an acceptable SRMR (0.066), and was therefore retained for DAG purposes.

Our findings indicated that the inverse association is driven by the gastrointestinal factor and is not attributable to a single trait in either cluster (gastrointestinal or metabolic). Additionally, this association is no longer significant when accounting for gastrointestinal alone. This should not be interpreted as metabolic disease being protective against MDD, but rather that MDD-relevant metabolic liability is largely shared within the gastrointestinal cluster, with a weak inverse conditional association when all four clusters are included.

In addition, to strengthen the robustness of the proposed model, we conducted an exploratory factor analysis (EFA) and examined differences in statistical power across disease clusters to assess potential sources of bias. First, the EFA supported the latent structure of the identified model, yielding a broadly similar loading pattern and providing data-driven support for the proposed structure. Second, the joint model shows that the gastrointestinal factor is most strongly associated with MDD. Power quantification indicated that the gastrointestinal cluster was moderately powered relative to the other clusters, indicating this association is not an artefact of power. Conversely, although the traits were well-powered, the immune cluster had the lowest statistical power comparatively. We further discuss considerations surrounding the immune cluster and these results (see next comment).

Methods: Line 329 to 334.

Exploratory Factor Analysis. We performed an exploratory factor analysis (EFA) to compare the number of factors and the loading patterns suggested by the data, without imposing the latent structure specified in the confirmatory model. We used the LDSC covariance matrix and the *fa* function in the *psych* R package[8], with oblique rotation (oblimin) and maximum likelihood (ml) settings. Factors with eigenvalues greater than one were extracted, and a loading cutoff of 0.30 was used to define strong loadings.

Supplementary Results: Lines 110 to 128.

2.6 Exploratory Factor Analysis

We conducted an exploratory factor analysis (EFA) to assess the latent structure and factor loadings. EFA indicated a five-factor model based on eigenvalues greater than one (Supplementary Figure 2a). The five-factor EFA model had similar metabolic, gastrointestinal, and immune groupings to the identified confirmatory model (Supplementary Table 4). The addition of the fifth factor split CVD traits into two clusters, creating subdivisions of the CVD cluster with only two traits loading onto each. The two CVD factors had the highest correlation across factors at 0.51 (Supplementary Figure 2b).

We next examined a four-factor exploratory solution to compare to the five-factor EFA (Supplementary Figure 2c; Supplementary Table 4). The four-factor EFA exhibited disease traits clustering closely with the confirmatory model. The traits showed clear groupings within the suggested factors, albeit with loadings below the cutoff in the immune cluster. When comparing the two EFA models, we observed minor improvements in fit indices with five factors (Supplementary Table 5; Five-factor: χ^2 = 2.298; fit.off = 0.985, df-RSMR = 0.056; Four-factor: χ^2 = 3.931, fit.off = 0.980, df-RSMR = 0.058). However, the four-factor model (BIC = -167.97) demonstrated superior fit to the five-factor model (BIC = -136.33), suggesting that the five-factor model did not provide sufficient improvement to justify the added model complexity. The fifth factor may represent a subdivision of CVD genetic liability rather than a broader physical disease domain.

Supplementary Results: Lines 313 to 333.

5.0 Power Quantification

We next explored differences in power across the disease clusters (Supplementary Table 19). First, we examined the power of the individual traits within the cluster using their h^2_{SNP} Z scores. Second, we investigated the genetic overlap among traits within each disease cluster, as reflected in the factor structure, i.e., the greater the shared genetic variance, the greater the variance captured by the latent factor. Together, the individual trait power and the extent of shared variance will influence the overall power of each physical disease cluster.

On average, the metabolic cluster had the highest h^2_{SNP} Z (mean = 16.83) and the strongest factor loadings (mean | λ | = 0.80), suggesting that this latent factor captures a relatively large proportion of shared variance across traits. Consistent with this stronger shared signal and trait power, the metabolic-MDD second-order factor yielded the largest number of loci (N = 537). Both CVD and gastrointestinal disease clusters showed broadly similar signal strength, as indicated by the input traits (CVD: mean h^2_{SNP} Z = 13.01; Gastro: mean h^2_{SNP} Z = 14.11) and genomic SEM parameters (CVD: mean | λ | = 0.69; Gastro: mean | λ | = 0.67). Accordingly, they yielded a similar number of loci in the multivariate GWAS (CVD: N = 172; Gastro: N = 170). Lastly, the immune cluster has the lowest average h^2_{SNP} Z (mean = 10.95) and the weakest factor loadings (mean | λ | = 0.35), indicating that the latent factor captured the least shared variance across traits. Nevertheless, the immune multivariate GWAS identified 140 loci, indicating that although the signal is comparatively weaker than the other clusters, it was not absent. Overall, these findings show differences in signal strength and shared variance across clusters, which reflect differences in locus discovery.

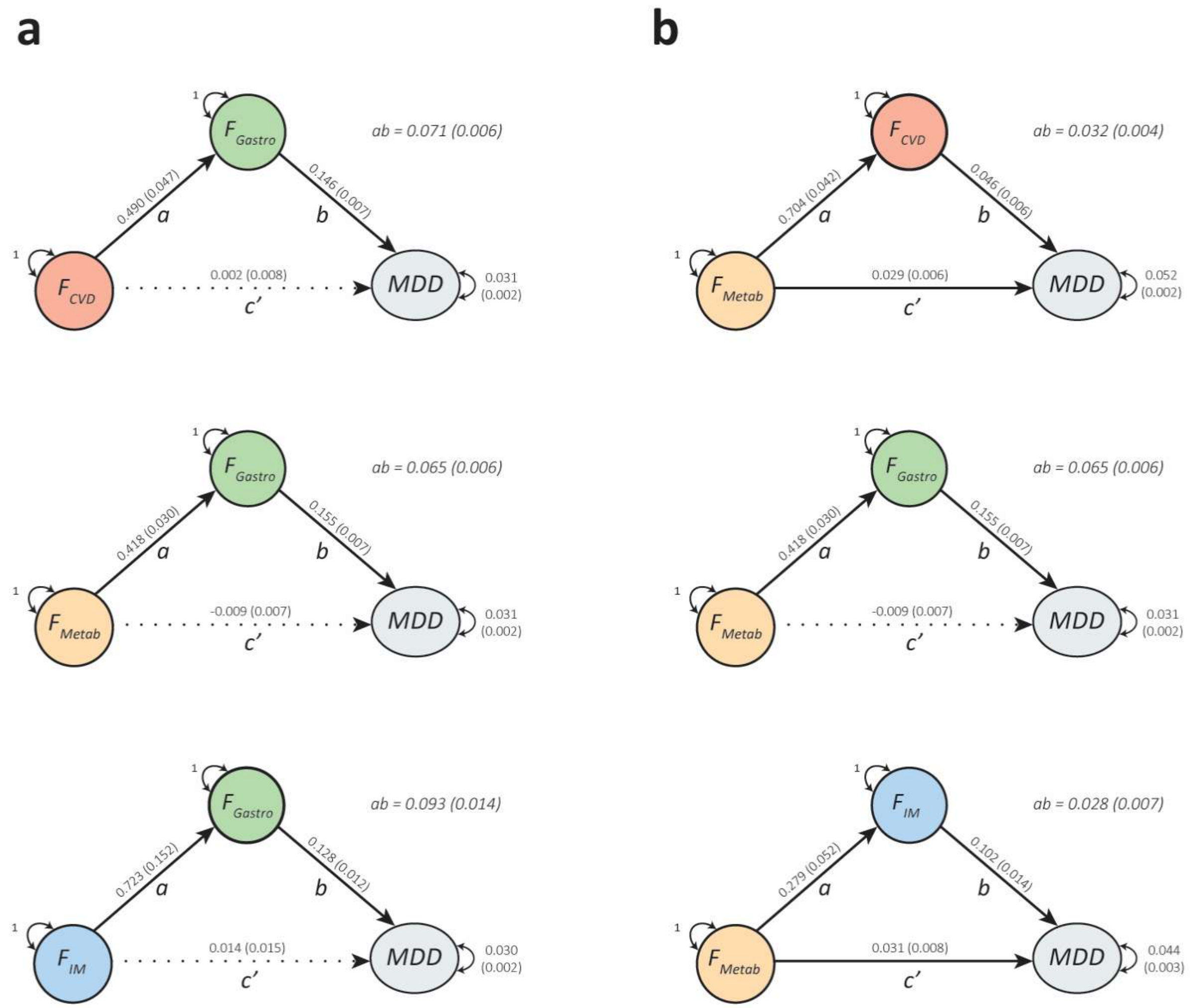

Supplementary Figure 3. Directed Acyclic Graphs (DAGs) examining gastrointestinal and metabolic clusters. a. DAGs investigating the impact of gastrointestinal cluster on the association between other disease clusters and MDD. **b.** DAGS exploring the driver of the negative association of the metabolic cluster and MDD. Dotted lines indicate an insignificant path (P > 0.05). The letters denote separate paths, the direct path (c') and the indirect path (a*b). Unstandardised estimates are described. Acronyms: Cardiovascular (CVD), metabolic (Metab), gastrointestinal (gastro), immune (IM), major depressive disorder (MDD).

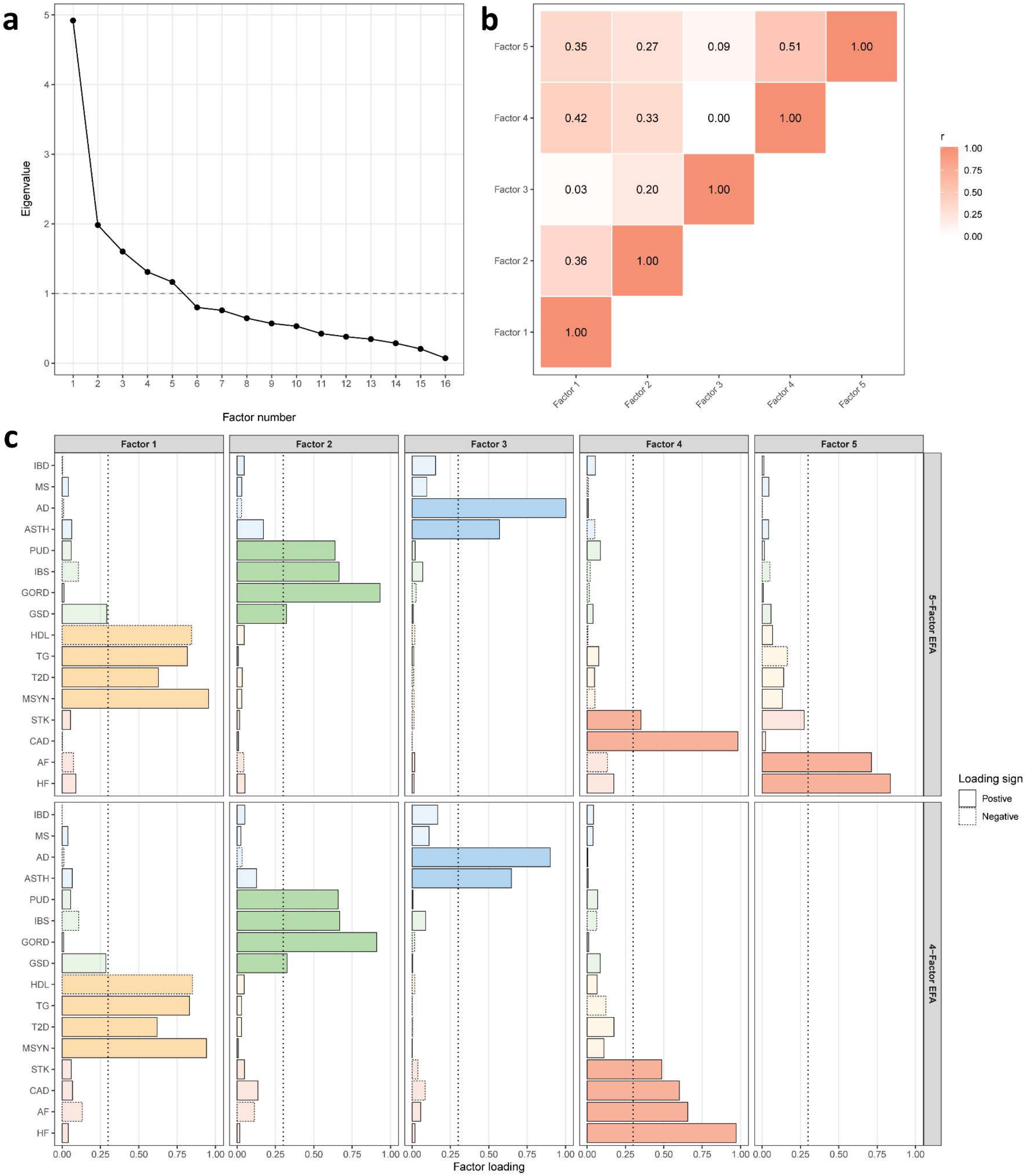

Supplementary Figure 2. Exploratory Factor Analysis (EFA) of the trait covariance structure. **a**. Scree plot of eigenvalues and the corresponding number of factors. **b.** The factor correlations of the five-factor exploratory solution. **c.** Comparing rotated factor loadings of a five-factor and four-factor EFA. Absolute factor loadings of each trait on the models are illustrated. Stronger loadings above the prespecified cutoff of 0.30 are emphasised, with negative loadings outlined in dotted lines. Acronyms: Atrial Fibrillation (AF), Coronary Artery Disease (CAD), Heart Failure (HF), Stroke (STK), Metabolic Syndrome (MSYN), Type 2 Diabetes (T2D), Triglycerides (TG), High-density lipoprotein cholesterol (HDL), Gallstone Disease (GSD), Gastro-oesophageal Reflux Disease (GORD), Irritable Bowel Syndrome (IBS), Peptic Ulcer Disease (PUD), Asthma (ASTH), Atopic Dermatitis (AD), Multiple Sclerosis (MS), Inflammatory Bowel Disease (IBD).

- The model proposed and investigated in several downstream analyses shows the gastrointestinal cluster as the one with the strongest association with MDD, with the immune cluster not showing independent association with MDD. However, when including traits with Z SNP-h2 > 4, the immune cluster explains the largest portion of MDD variance and has the strongest correlation with MDD, while the gastrointestinal group has no longer independent association. The authors discard this model with the justification that is shows lower power, and that "the immune cluster was heavily manipulated". This choice should be better discussed, better explaining in which ways "the immune cluster was heavily manipulated", as the model fit looks similar to other latent factors and the strong correlation between ASTH and AD was present also in the model including only traits with Z SNP-h2 > 7. A further reason to address this point is that pathogenetic mechanisms involving the immune system have been reported in MDD (e.g., PMID 31238086, 34724041, 32553197) and that the instability in the effect of the gastrointestinal factor between the two approaches may rise some concerns.

We have added more discussion on why the analysis using a lower threshold for the factor loadings was not continued, specifically noting model parameters indicating model instability.

Supplementary Results: Lines 222 to 237

The lower Z score threshold was not retained for several reasons. Firstly, the CFI model fit (CFI = 0.848) fell below the 0.90 threshold. Second, we observed high standard errors for the residual variances of the lower-powered traits (4 < Z < 7) (IDA SE = 0.19; PAD SE = 0.14; HD SE = 0.20; PSV SE = 0.18). Importantly, this indirectly inflated the standard errors of the regression coefficients for the immune (SE = 0.24) and gastrointestinal (SE = 0.19) clusters on MDD, indicating imprecise estimates. Third, the immune latent factor loadings were skewed towards Hashimoto's disease (HD) and psoriasis (PSV), distorting the loadings and emphasising the contribution of the weaker traits relative to the more reliable, well-powered immune traits.

Overall, these considerations demonstrate that the lower-powered traits, acting as influential outliers, disproportionately influence correlations in modelling. Specifically,

the immune cluster was distorted, as evidenced by the reduced factor loadings of other well-powered traits and inflating the immune group's independent association with MDD. This is further evident when compared to the h^2_{SNP} Z > 7 models. Correlations between the immune and other factor groups are reduced, standard errors stabilise, all factor loadings of the immune traits increase, and the independent association of the immune factor with MDD attenuates, thereby enabling the independent associations of cardiovascular and gastrointestinal factors to emerge.

We further consider factors and limitations that influenced the immune cluster and its results. Additional discussion has been added to the manuscript.

Manuscript: Lines 257 to 272

Lastly, the immune cluster was not independently associated with MDD in the joint model. This does not implicate immune mechanisms as unrelated to MDD; rather, this cluster's association with MDD is shared with other factors, particularly the gastrointestinal factor, which could reflect the strong link between gastrointestinal disorders and immune mechanisms[1]. However, we note several limitations of the immune cluster that may influence these findings. This cluster had the smallest shared variance among the individual traits, as evidenced by its weaker genetic correlations, factor loadings, and trait residual variance, consistent with a less uniform shared signal. A potential cause is the heterogeneous nature of immune disorders, with various subtypes, immune responses, and effects on different tissues and organs. Cross-disorder genetic analyses have revealed distinct clusters of genetic associations in immune-mediated diseases[2] as well as psychiatric factors associated with immune subtypes to different degrees[3]. Additionally, the MHC region was removed from genomic SEM and LDSC due to the complex LD structure, which may also influence the results of the immune cluster, given that HLA type is often the strongest common genetic risk factor for immune-driven disorders. With larger sample sizes for immune traits, future work should examine the various immune subtypes and the influence of the MHC region on physical disease-MDD comorbidities.

- The authors suggest that the gastrointestinal cluster represents the group of physical diseases with the strongest association with MDD. Looking at several analyses, gastrointestinal diseases also seem the group with the greatest shared genetics with the other diseases of interest, e.g., looking at the associations in figure 3 and the number of non-shared ACAT hits in figure 5A (gastro-MDD has 88/406, while other diseases-MDD pairs all have higher proportion of non-shared hits). The pheWAS analysis also show notable overlap of the identified traits among gastro-MDD and the others disease-MDD pairs, as well as cell-type specific gene associations, suggesting non-specific mechanisms. The interpretation of these findings should be expanded, considering also that the emergence of the gastrointestinal factor looked relatively unexpected, particularly compared to cardiovascular and metabolic factors, and it is not clear what could be driving this result.

The results demonstrated that the gastrointestinal cluster had the highest genetic overlap with MDD. We note that this is likely not due to gastrointestinal pathology as the primary driver and is consistent with psychophysiological gut-brain liability. In the joint model, attenuation of other physical disease clusters indicates that their association with MDD is largely shared with correlating clusters, with particular emphasis on the gastrointestinal cluster. This suggests the convergence of immune/metabolic/CVD pathways is captured in a broader gut-brain axis.

The lack of specificity in the gastrointestinal disorders is consistent with the degree of overlap with MDD and the other cluster. Gastrointestinal phenotypes, particularly functional subtypes, are closely aligned with depression liability. This clustering can overlap with both the physical and affective components of MDD. Given the amount of genetic overlap with MDD, a smaller unique gastrointestinal component is expected.

From a biological/molecular perspective, high overlap between MDD and gastrointestinal disorders is reasonable. The enteric nervous system plays a key role in the brain-gut axis, acting as a bidirectional bridge between gut function and central mood regulation. This system contains pathways closely tied to stress response and somatic symptoms that commonly co-occur with MDD. A gastrointestinal latent factor can plausibly capture a gut-brain component of MDD-related biology. Additionally, drug

categories used to treat functional gastrointestinal disorders have genetic targets overlapping with those for psychiatric treatments. We observed enrichment of these targets and identified them as potential markers of shared pathways that manifest in both the ENS and the CNS, particularly the D2 receptor.

The discussion section has been further expanded to provide further interpretation of the emergence of the gastrointestinal cluster, highlighting the biological mechanisms and potential manifestations of shared liability between gastrointestinal-MDD.

Manuscript: Lines 200 to 217

The enteric nervous system (ENS) is a key component of the gut-brain relationship. The ENS and CNS have shared neurotransmission, signalling cascades, and structural features, which often result in CNS pathology emerging in enteric systems[22]. We observed that gastrointestinal-MDD shared genetic liability was associated with functional gastrointestinal disease, gene regulatory signals in digestive tissues, and drug classes that enhance gut motility (e.g., propulsives). Together with identified neuronal gene sets, these findings suggest that neural mechanisms of gut function may contribute to this genetic overlap and point to ENS-related manifestations. We observed the dopaminergic system as an enriched neurotransmitter system, with particular emphasis on the D2 receptor in both MDD and gastrointestinal liability. This receptor has been shown to be a key communicator between the gut, brain and microbiota[23], is associated with multiple gastrointestinal diseases[24], and shows digestive benefits when targeted with medication[24]. For example, many gastrointestinal treatments (e.g., Domperidone[24]) are D2 receptor inhibitors or antagonists for which we show upregulated expression in gastrointestinal tissues. Additionally, the ENS is tightly coupled with immune-related mechanisms[25] which may contribute to the observed attenuation of the MDD-immune associations. Further research should examine the CNS-ENS relationship in MDD, including immune responses, and the manifestations of MDD-physical disease liability in neurotransmission, particularly dopaminergic pathways.

- Latent causal variable analysis did not support causality (supplementary results), however this is not clear in the main manuscript text. Given the risk that CAUSE results are inflated, statements about causal effects should be much more cautious in the main manuscript text.

Thank you for pointing this out. We have adjusted this in the main manuscript.

Manuscript: Lines 125 to 134

We further explored MDD and the gastrointestinal cluster to elucidate whether this strong relationship arises due to genetic risk acting through the same pathway (horizontal pleiotropy) or acting through one trait that influences the other (vertical pleiotropy) using the latent causal variable (LCV)[26] and Causal Analysis Using Summary Effect (CAUSE)[27] models. We found evidence for a bidirectional causal effect (vertical pleiotropy) between the MDD genetic liability and the gastrointestinal cluster, with a slightly stronger effect in the direction from MDD to the gastrointestinal cluster (Section 8.2; Supplementary Figure 10). However, due to high levels of correlated pleiotropy, CAUSE results may be inflated. This analysis should be interpreted cautiously, and further work is needed to resolve the true extent of this relationship.

- The GWAS of MDD used in this study included population-based samples (PMID 37464041), e.g., iPSYCH, where different diagnoses can be reported for the same individual over time and there is no structured assessment. The possible implications of this should be discussed, including the impact on the results.

We acknowledge this is a limitation of GWAS and minimal phenotyping. Specifically for this manuscript, this limitation may dilute specific subtypes of depression relating to physical disease comorbidities. We have added this limitation to the manuscript and summarised the potential impact on the results.

Manuscript: Lines 236 to 241

Third, broad phenotypic definitions used in the MDD GWAS will increase genetic heterogeneity and dilute disorder-specific signals and MDD subtypes[28] (e.g., inflammatory depression), while also inflating trait overlap with proximal or related symptoms (e.g., functional gastrointestinal symptoms). Consequently, the physical disease-MDD associations must be interpreted pertaining to the cohort's general ascertainment rather than a strict, clinically uniform MDD phenotype.

- The inclusion of GWASs of European ancestry only is an important limitation.

We note this important limitation in the discussion. As this analysis requires many well-powered traits per cluster, performing it in other ancestries is not currently possible due to data availability.

Minor comments:

- "[...] and identify tissues with a significant effect across tissues" should likely be features (or genes) with a significant effect across tissues.

- "anxiolytics (drugs used to treat anxiety and/or depression)": anxiolytics have no indication for the treatment of depression.

Thank you for spotting these errors. They have been amended.

References

1. Layunta, E., Mesonero, J. E. & Latorre, E. Guardians at the Gate: Immune System in Gastrointestinal Diseases. *Int J Mol Sci* **25**, 5933 (2024).
2. Demela, P., Pirastu, N. & Soskic, B. Cross-disorder genetic analysis of immune diseases reveals distinct gene associations that converge on common pathways. *Nat Commun* **14**, 2743 (2023).
3. Breunig, S. *et al.* Examining the genetic links between clusters of immune-mediated diseases and psychiatric disorders. *Transl Psychiatry* **15**, 252 (2025).
4. Ali, K. M., Wonnerth, A., Huber, K. & Wojta, J. Cardiovascular disease risk reduction by raising HDL cholesterol – current therapies and future opportunities. *Br J Pharmacol* **167**, 1177–1194 (2012).
5. Klancic, T., Woodward, L., Hofmann, S. M. & Fisher, E. A. High density lipoprotein and metabolic disease: Potential benefits of restoring its functional properties. *Mol Metab* **5**, 321–327 (2016).
6. Ratheesh, R., Ulrich, M. T., Ghozy, S., Al-Jaboori, M. & Nayak, S. S. The association between diabetes and gallstones: a nationwide population-based cohort study. *Prz Gastroenterol* **18**, 292–299 (2023).
7. Ata, N. *et al.* The metabolic syndrome is associated with complicated gallstone disease. *Can J Gastroenterol* **25**, 274–276 (2011).
8. Revelle, W. *Psych: Procedures for Psychological, Psychometric, and Personality Research*. (Northwestern University, Evanston, Illinois, 2026).
9. Bulik-Sullivan, B. K. *et al.* LD Score regression distinguishes confounding from polygenicity in genome-wide association studies. *Nat Genet* **47**, 291–295 (2015).

10. Foote, I. F. *et al.* The shared genetic architecture of modifiable risk for Alzheimer's disease: a genomic structural equation modelling study. *Neurobiol Aging* **117**, 222–235 (2022).
11. Lawrence, J. M. *et al.* Shared Genetic Liability across Systems of Psychiatric and Physical Illness. *Nat Commun* **17**, 2993 (2026).
12. Berk, M. *et al.* Comorbidity between major depressive disorder and physical diseases: a comprehensive review of epidemiology, mechanisms and management. *World Psychiatry* **22**, 366–387 (2023).
13. Bergstedt, J. *et al.* Distinct biological signature and modifiable risk factors underlie the comorbidity between major depressive disorder and cardiovascular disease. *Nat Cardiovasc Res* **3**, 754–769 (2024).
14. Ghanei Gheshlagh, R., Parizad, N. & Sayehmiri, K. The Relationship Between Depression and Metabolic Syndrome: Systematic Review and Meta-Analysis Study. *Iran Red Crescent Med J* **18**, e26523 (2016).
15. Chourpiliadis, C. *et al.* Metabolic Profile and Long-Term Risk of Depression, Anxiety, and Stress-Related Disorders. *JAMA Network Open* **7**, e244525 (2024).
16. Gong, W. *et al.* Role of the Gut-Brain Axis in the Shared Genetic Etiology Between Gastrointestinal Tract Diseases and Psychiatric Disorders: A Genome-Wide Pleiotropic Analysis. *JAMA Psychiatry* **80**, 360 (2023).
17. Penninx, B. W. J. H. *et al.* Immuno-metabolic depression: from concept to implementation. *Lancet Reg Health Eur* **48**, 101166 (2024).
18. Singh, A., Kishore, P. S. & Khan, S. From Microbes to Myocardium: A Comprehensive Review of the Impact of the Gut-Brain Axis on Cardiovascular Disease. *Cureus* **16**, e70877.

19. Pan, Y., Bu, T., Deng, X., Jia, J. & Yuan, G. Gut microbiota and type 2 diabetes mellitus: a focus on the gut-brain axis. *Endocrine* **84**, 1–15 (2024).
20. dos Santos, A. & Galiè, S. The Microbiota–Gut–Brain Axis in Metabolic Syndrome and Sleep Disorders: A Systematic Review. *Nutrients* **16**, 390 (2024).
21. Khawar, M. M. *et al.* The Gut-Brain Axis in Autoimmune Diseases: Emerging Insights and Therapeutic Implications. *Cureus* **15**, e48655.
22. Rao, M. & Gershon, M. D. The bowel and beyond: the enteric nervous system in neurological disorders. *Nat Rev Gastroenterol Hepatol* **13**, 517–528 (2016).
23. Xie, Y.-A., Kong, J.-D., Li, S. & Wei, D.-F. What is the Impact of Dopamine D2 Receptor in the Brain-Gut Axis? A Narrative Review of the Mechanism Based on Gut Microbiota in Modulating Emotion and Behavior. *Alpha Psychiatry* **26**, 39226 (2025).
24. Lu, X. *et al.* Research progress on the roles of dopamine and dopamine receptors in digestive system diseases. *J Cell Mol Med* **28**, e18154 (2024).
25. Wang, H., Foong, J. P. P., Harris, N. L. & Bornstein, J. C. Enteric neuroimmune interactions coordinate intestinal responses in health and disease. *Mucosal Immunol* **15**, 27–39 (2022).
26. O'Connor, L. J. & Price, A. L. Distinguishing genetic correlation from causation across 52 diseases and complex traits. *Nat Genet* **50**, 1728–1734 (2018).
27. Morrison, J., Knoblauch, N., Marcus, J. H., Stephens, M. & He, X. Mendelian randomization accounting for correlated and uncorrelated pleiotropic effects using genome-wide summary statistics. *Nat Genet* **52**, 740–747 (2020).
28. Flint, J. The genetic basis of major depressive disorder. *Mol Psychiatry* **28**, 2254–2265 (2023).
